# Preliminary SAR of Novel Pleuromutilin–Polyamine Conjugates

**DOI:** 10.3390/microorganisms11112791

**Published:** 2023-11-17

**Authors:** Kenneth Sue, Melissa M. Cadelis, Kerrin Hainsworth, Florent Rouvier, Marie-Lise Bourguet-Kondracki, Jean Michel Brunel, Brent R. Copp

**Affiliations:** 1 School of Chemical Sciences, The University of Auckland, Private Bag 92019, Auckland 1142, New Zealand; 2 School of Medical Sciences, The University of Auckland, Private Bag 92019, Auckland 1142, New Zealand; 3 Membranes et Cibles Thérapeutiques, INSERM, Aix-Marseille Universite, 27 bd Jean Moulin, 13385 Marseille, France; 4 Laboratoire Molécules de Communication et Adaptation des Micro-organismes, UMR 7245 CNRS, Muséum National d’Histoire Naturelle, 57 rue Cuvier (C.P. 54), 75005 Paris, France

**Keywords:** polyamine, pleuromutilin, antimicrobial, antibiotic enhancement, membrane disruption

## Abstract

While pleuromutilin (**1**) and its clinically available derivatives (**2**–**6**) are highly effective against Gram-positive bacteria, they remain inactive against many pathogenic Gram-negative bacteria due to the efflux pump AcrAB-TolC. In an effort to broaden the spectrum of activity of pleuromutilin (**1**), we developed a series of novel pleuromutilin–polyamine conjugates (**9a**–**f**) which exhibited promising intrinsic antimicrobial properties, targeting both Gram-positive and Gram-negative bacteria, including *Staphylococcus aureus*, methicillin-resistant *S. aureus* (MRSA), and *Escherichia coli*, along with the fungal strain *Cryptococcus neoformans*, and were devoid of cytotoxic and hemolytic properties with the exception of one conjugate. Furthermore, this series displayed moderate to low antibiotic potentiation of legacy antibiotics doxycycline and erythromycin, with three conjugates enhancing the activity four-fold in combination with doxycycline. In comparison to pleuromutilin (**1**) and tiamulin (**2**), one of the conjugates exhibited an expanded spectrum of activity, including Gram-negative bacteria and fungi, making it a promising option for combating microbial infections.

## 1. Introduction

The rise in antimicrobial resistance, caused by the excessive use of antibiotics and a lack of investment towards the discovery of new antibiotics, has resulted in a growing number of challenging microbial infections [1,2,3,4,5,6]. The discovery of new antibiotics is one avenue for overcoming resistant microbes, while another is the discovery of compounds with little or no antimicrobial activity which can be used in combination with legacy antibiotics to restore their activities [6].

**Figure 1:**
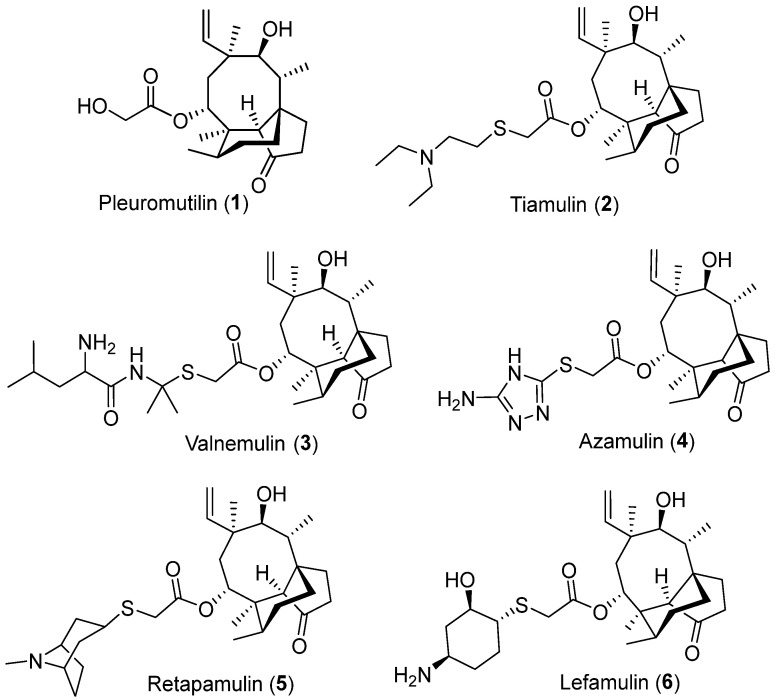
Structures of pleuromutilin (**1**) and its derivatives (**2**–**6**).

Upon its discovery in 1951, the diterpenoid antibiotic pleuromutilin (**1**), from the fungi *Pleurotus mutilus* (currently known as *Clitopilus scyphoides*) and *P. passeckerianus* (*C. passeckerianus*), was reported to exhibit bactericidal activity against *Staphylococcus aureus* and moderate activity against *Mycobacterium smegmatis* but showed no effect against *Escherichia coli* [7,8,9,10]. However, the exact structure of pleuromutilin (**1**) (Figure 1) remained unknown until a decade after its initial discovery, after which researchers synthesized derivatives focusing on substituting the hydroxyl group at C-22 [8,10,11]. This led to the development of tiamulin (**2**), an analogue achieved through substitution with sulphonate esters, which gained approval for veterinary use in 1979. While these derivatives, including the second-generation pleuromutilin antibiotic valnemulin (**3**), which boasted improved potency, were successful in veterinary applications, pharmaceutical companies aimed to develop pleuromutilin antibiotics for human use [8,10,11]. In 1982, progress was made towards this goal with azamulin (**4**) entering phase I clinical trials, though it failed to pass due to poor bioavailability [8,12]. Nevertheless, the breakthrough came with retapamulin (**5**), a potent pleuromutilin analogue approved by the FDA in 2007 for topical infections, sparking renewed interest in pleuromutilin and its derivatives [8,10,13]. More recently, lefamulin (**6**), another pleuromutilin derivative, successfully passed phase III clinical trials for bacterial pneumonia and improved upon retapamulin (**5**) as it could be administered orally or intravenously [14,15].

Lefamulin displayed potent antibacterial activity against aerobic Gram-positive bacteria, proving effective against challenging strains such as methicillin-resistant *S. aureus* (MRSA), vancomycin-intermediate *S. aureus* (VISA), heterogeneous VISA (hVISA), vancomycin-resistant *S. aureus* (VRSA), penicillin-resistant *Streptococcus pneumoniae* (PRSP), multidrug-resistant *S. pneumoniae,* and vancomycin-resistant *Enterococcus faecium* (VRE) [10,16,17,18]. Additionally, it exhibited favorable to moderate activity against fastidious Gram-negative bacteria, including *Haemophilus influenza* and *Moraxella catarrhalis*. However, like its predecessors, no activity was observed against non-fastidious Gram-negative bacteria [10,16,17,18].

The lack of activity of this structural class against Gram-negative bacteria was attributed to the expression of the efflux pump AcrAB-TolC, a tripartite efflux pump which exports small molecules from the bacterial cell, reducing drug efficacy [10,19,20]. Encouragingly, a derivative of pleuromutilin showed promise against an *E. coli* strain when tested in the presence of the efflux pump inhibitor Phe-Arg-β-naphthylamide [10], indicating that inhibiting AcrAB-TolC could potentially enable pleuromutilin to be used in the treatment of Gram-negative bacterial infections.

SAR studies show that pleuromutilins with thioether or basic group linkers have enhanced antibacterial activity. Recent research suggests that increasing primary amines and positive charges in antibiotics can improve their uptake and accumulation in Gram-negative bacteria, potentially broadening their spectrum beyond Gram-positives. In our previous work on α,ω polyamines with disubstituted lipophilic head groups, we have successfully demonstrated examples that inhibit AcrAB-TolC [21]. Thus, the present study aims to synthesize and evaluate a series of pleuromutilin–polyamine conjugates bearing varying polyamine chain lengths for their intrinsic antimicrobial activity and their ability to enhance the activity of doxycycline and erythromycin against Gram-negative bacteria.

## 2. Materials and Methods

### 2.1. Chemical Synthesis General Methods

See Supplementary File [22,23,24].

### 2.2. Synthesis of Compounds

#### 2.2.1. Pleuromutilin 22-*O*-Tosylate (**8**)

A solution of pleuromutilin (**1**) (0.50 g, 1.3 mmol), toluenesulfonylchloride (0.30 g, 1.6 mmol), and DMAP (0.48 g, 3.9 mmol) in anhydrous CH_2_Cl_2_ was stirred at 0 °C for 4 h under N_2_ atmosphere. The reaction was quenched with 1 N HCl and extracted twice with EtOAc. The combined organic layers were then washed with sat. *aq*. NaHCO_3_, dried with MgSO_4_, and concentrated under reduced pressure. The crude product was purified by diol-bonded silica gel column chromatography (20–80% EtOAc/petroleum ether) to afford (**8**) as a white foam (0.39 g, 57%). ^1^H and ^13^C NMR data agreed with those reported in the literature [25].

#### 2.2.2. Tiamulin (**2**)

A solution of pleuromutilin 22-*O*-tosylate (**8**) (0.130 g, 0.244 mmol) and KI (0.080 g, 0.482 mmol) in MeCN (10 mL) was stirred at 70 °C under an N_2_ atmosphere for 30 min. A solution of 2-diethylaminoethane thiol hydrochloride (0.046 g, 0.271 mmol) and DIPEA (0.26 mL, 1.49 mmol) in anhydrous MeCN (2 mL) was then added and the reaction stirred for a further 2 h. The solvent was removed under reduced pressure, to which was added CH_2_Cl_2_ (30 mL), and the organic phase was washed with sat. *aq*. NaHCO_3_ (2 × 30 mL) and H_2_O (2 × 30 mL) and dried over anhydrous Na_2_SO_4_, and the solvent was removed again under reduced pressure. The crude product was purified by diol-bonded silica gel column chromatography (75–100% EtOAc/hexane followed by 100% MeOH) to afford **2** as a pale orange foam (0.057 g, 47%). R*_f_* (silica gel, 100% EtOAc) 0.44; ${\left[\alpha \right]}_{D}^{23.3}=+$50.6 (*c* 0.195, CH_2_Cl_2_); IR (ATR) ν_max_ 3444, 2929, 1721, 1455, 1277, 1115 cm^−1^; ^1^H NMR (CDCl_3_, 400 MHz) *δ* 6.47 (1H, dd, *J* = 17.4, 11.0 Hz, H-19), 5.74 (1H, d, *J* = 8.5 Hz, H-14), 5.33 (1H, dd, *J* = 11.0, 1.5 Hz, H_2_-20a), 5.19 (1H, dd, *J* = 17.4, 1.5 Hz, H_2_-20b), 3.35 (1H, d, *J* = 6.1 Hz, H-11), 3.16 (2H, s, H_2_-22), 2.68 (4H, s, H_2_-23, H_2_-24), 2.53 (4H, q, *J* = 7.1 Hz, 2H_2_-25), 2.38–2.32 (1H, m, H-10), 2.25–2.16 (2H, m, H_2_-2), 2.11–2.05 (2H, m, H-4, H_2_-13a), 1.79–1.74 (1H, m, H_2_-8a), 1.69–1.60 (2H, m, H_2_-1a, H-6), 1.57–1.50 (1H, m, H_2_-7a), 1.48–1.41 (1H, m, H_2_-1b), 1.45 (3H, s, H_3_-15), 1.39–1.32 (2H, m, H_2_-7b, H_2_-13b), 1.16 (3H, s, H_3_-18), 1.15–1.08 (1H, m, H_2_-8b), 1.02 (6H, t, *J* = 7.2 Hz, 2H_3_-26), 0.87 (3H, d, *J* = 7.2 Hz, H_3_-17), 0.73 (3H, d, *J* = 6.9 Hz, H_3_-16), OH signal not observed; ^13^C NMR (CDCl_3_, 100 MHz) *δ* 217.2 (C-3), 169.1 (C-21), 139.2 (C-19), 117.3 (C-20), 74.7 (C-11), 69.4 (C-14), 58.3 (C-4), 52.0 (C-24), 46.9 (C-25), 45.6 (C-9), 44.9 (C-13), 44.0 (C-12), 41.9 (C-5), 36.9 (C-6), 36.1 (C-10), 34.63 (C-22), 34.58 (C-2), 30.5 (C-8), 29.4 (C-23), 27.0 (C-7), 26.5 (C-18), 25.0 (C-1), 17.0 (C-16), 15.0 (C-15), 11.6 (C-17), 11.2 (C-26); (+)-HRESIMS *m/z* 494.3287 [M + H]^+^ (calculated for C_28_H_48_NO_4_S, 494.3299).

#### 2.2.3. *N*^1^,*N*^1^′-(Butane-1,4-diyl)bis(*N*^3^-(2-(((3a*R*,4*R*,5*R*,7*S*,8*S*,9*R*,9a*S*,12*R*)-8-hydroxy-4,7,9,12-tetramethyl-3-oxo-7-vinyldecahydro-4,9a-propanocyclopenta [8]annulen-5-yl)oxy)-2-oxoethyl)propane-1,3-diaminium) 2,2,2-trifluoroacetate (**9a**)

Following general procedure A, the reaction of pleuromutilin 22-*O*-tosylate (**8**) (0.025 g, 0.047 mmol), KI (0.009 g, 0.054 mmol), DIPEA (0.025 mL, 0.144 mmol), and di-*tert*-butyl butane-1,4-diylbis((3-aminopropyl)carbamate) (**7a**) (0.009 g, 0.024 mmol) afforded bis((3a*R*,4*R*,5*R*,7*S*,8*S*,9*R*,9a*S*,12*R*)-8-hydroxy-4,7,9,12-tetramethyl-3-oxo-7-vinyldecahydro-4,9a-propanocyclopenta [8]annulen-5-yl) 7,12-bis(*tert*-butoxycarbonyl)-3,7,12,16-tetraazaoctadecanedioate (0.019 g, 72%) as a yellow oil. Following general procedure B, a sub-sample of the protected product (0.010 g, 0.009 mmol) was reacted with TFA in CH_2_Cl_2_ to afford the tetra-TFA salt **9a** (0.008 g, 65%) as a yellow oil. ${\left[\alpha \right]}_{D}^{18.8}=+11$ (*c* 0.1, MeOH); R*_f_* (RP-18, 10% *aq* HCl:MeOH 1:3) 0.57; IR (ATR) ν_max_ 2922, 1732, 1671, 1420, 1179, 1121, 1020, 914, 833, 798, 720 cm^−1^; ^1^H NMR (DMSO-*d*_6_, 400 MHz) δ 9.34 (4H, br s, NH_2_-23), 8.82 (4H, br s, NH_2_-27), 6.14 (2H, dd, *J* = 17.8, 11.2 Hz, H-19), 5.63 (2H, d, *J* = 8.2 Hz, H-14), 5.14 (2H, dd, *J* = 17.8, 1.4 Hz, H_2_-20a), 5.06 (2H, dd, *J* = 11.2, 1.1 Hz, H_2_-20b), 4.05 (2H, d, *J* = 17.9 Hz, H_2_-22a), 3.86 (2H, d, *J* = 17.4 Hz, H_2_-22b), 3.45 (2H, d, *J* = 8.2 Hz, H-11), 3.02–2.95 (8H, m, H_2_-24, H_2_-26), 2.95–2.89 (4H, m, H_2_-28), 2.46 (2H, s, H-4), 2.24–1.95 (12H, m, H_2_-2, H-10, H_2_-13a, H_2_-25), 1.69–1.62 (8H, m, H_2_-1b, H_2_-8a, H_2_-29), 1.56–1.50 (2H, m, H-6), 1.40–1.23 (8H, m, H_2_-1a, H_2_-7, H_2_-13b), 1.38 (6H, s, H_3_-15), 1.08 (6H, s, H_3_-18), 1.03–0.99 (2H, m, H_2_-8b), 0.83 (6H, d, *J* = 6.8 Hz, H_3_-17), 0.65 (6H, d, *J* = 6.8 Hz, H_3_-16); ^13^C NMR (DMSO-*d*_6_, 100 MHz) δ 217.0 (C-3), 165.6 (C-21), 140.8 (C-19), 115.5 (C-20), 72.4 (C-11), 71.1 (C-14), 57.0 (C-4), 47.4 (C-22), 46.1 (C-28), 44.9 (C-9), 44.2 (C-12), 44.0 (C-24/C-26), 43.8 (C-24/C-26), 43.3 (C-13), 41.5 (C-5), 36.5 (C-10), 36.2 (C-6), 34.0 (C-2), 30.0 (C-8), 28.5 (C-18), 26.6 (C-7), 24.4 (C-1), 22.7 (C-29), 22.1 (C-25), 16.2 (C-16), 14.4 (C-15), 11.6 (C-17); (+)-HRESIMS [M + H]^+^ *m/z* 923.6834 (calculated for C_54_H_91_N_4_O_8_, 923.6831).

#### 2.2.4. *N*^1^,*N*^1^′-(Hexane-1,6-diyl)bis(*N*^3^-(2-(((3a*R*,4*R*,5*R*,7*S*,8*S*,9*R*,9a*S*,12*R*)-8-hydroxy-4,7,9,12-tetramethyl-3-oxo-7-vinyldecahydro-4,9a-propanocyclopenta [8]annulen-5-yl)oxy)-2-oxoethyl)propane-1,3-diaminium) 2,2,2-trifluoroacetate (**9b**)

Following general procedure A, the reaction of pleuromutilin 22-*O*-tosylate (**8**) (0.050 g, 0.094 mmol), KI (0.017 g, 0.103 mmol), DIPEA (0.049 mL, 0.281 mmol), and di-*tert*-butyl hexane-1,6-diylbis((3-aminopropyl)carbamate) (**7b**) (0.020 g, 0.046 mmol) afforded bis((3a*R*,4*R*,5*R*,7*S*,8*S*,9*R*,9a*S*,12*R*)-8-hydroxy-4,7,9,12-tetramethyl-3-oxo-7-vinyldecahydro-4,9a-propanocyclopenta [8]annulen-5-yl) 7,14-bis(*tert*-butoxycarbonyl)-3,7,14,18-tetraazaicosanedioate (0.028 g, 52%) as a yellow oil. Following general procedure B, a sub-sample of the protected product (0.014 g, 0.012 mmol) was reacted with TFA in CH_2_Cl_2_ to afford, after chromatography, the tetra-TFA salt **9b** (0.009 g, 53%) as a yellow oil. ${\left[\alpha \right]}_{D}^{19.9}=+13$ (*c* 0.1, MeOH); R*_f_* (RP-18, 10% *aq* HCl:MeOH 1:3) 0.50; IR (ATR) ν_max_ 2928, 1739, 1671, 1464, 1413, 1199, 1180, 1131, 798, 721 cm^−1^; ^1^H NMR (DMSO-*d*_6_, 400 MHz) δ 9.32 (4H, br s, NH_2_-23), 8.69 (4H, br s, NH_2_-27), 6.15 (2H, dd, *J* = 17.8, 11.2 Hz, H-19), 5.63 (2H, d, *J* = 8.3 Hz, H-14), 5.14 (2H, dd, *J* = 17.5, 1.5 Hz, H_2_-20a), 5.07 (2H, dd, *J* = 11.1, 1.3 Hz, H_2_-20b), 4.61 (2H, br s, OH-11), 4.05 (2H, d, *J* = 16.9 Hz, H_2_-22a), 3.85 (2H, d, *J* = 16.7 Hz, H_2_-22b), 3.45 (2H, obscured by H_2_O, H-11), 3.00–2.93 (8H, m, H_2_-24, H_2_-26), 2.90–2.84 (4H, m, H_2_-28), 2.46 (2H, s, H-4), 2.24–1.94 (12H, m, H_2_-2, H-10, H_2_-13a, H_2_-25), 1.70–1.62 (8H, m, H_2_-1b, H_2_-8a, H_2_-29), 1.60–1.51 (2H, m, H-6), 1.46–1.36 (6H, m, H_2_-7, H_2_-13b), 1.38 (6H, s, H_3_-15), 1.33–1.24 (6H, s, H_2_-1a, H_2_-30), 1.08 (6H, s, H_3_-18), 1.03–1.00 (2H, m, H_2_-8b), 0.83 (6H, d, *J* = 6.9 Hz, H_3_-17), 0.65 (6H, d, *J* = 7.0 Hz, H_3_-16); ^13^C NMR (DMSO-*d*_6_, 100 MHz) δ 217.0 (C-3), 165.6 (C-21), 140.8 (C-19), 115.5 (C-20), 72.4 (C-11), 71.1 (C-14), 57.0 (C-4), 47.4 (C-22), 46.7 (C-28), 44.9 (C-9), 44.2 (C-12), 44.0 (C-24/C-26), 43.8 (C-24/C-26), 43.2 (C-13), 41.5 (C-5), 36.4 (C-10), 36.2 (C-6), 34.0 (C-2), 30.0 (C-8), 28.5 (C-18), 26.6 (C-7), 25.8 (C-30), 25.4 (C-29), 24.4 (C-1), 22.1 (C-25), 16.2 (C-16), 14.4 (C-15), 11.6 (C-17); (+)-HRESIMS [M + H]^+^ *m/z* 951.7142 (calculated for C_56_H_95_N_4_O_8_, 951.7144).

#### 2.2.5. *N*^1^,*N*^1^′-(Heptane-1,7-diyl)bis(*N*^3^-(2-(((3a*R*,4*R*,5*R*,7*S*,8*S*,9*R*,9a*S*,12*R*)-8-hydroxy-4,7,9,12-tetramethyl-3-oxo-7-vinyldecahydro-4,9a-propanocyclopenta [8]annulen-5-yl)oxy)-2-oxoethyl)propane-1,3-diaminium) 2,2,2-trifluoroacetate (**9c**)

Following general procedure A, the reaction of pleuromutilin 22-*O*-tosylate (**8**) (0.050 g, 0.094 mmol), KI (0.017 g, 0.103 mmol), DIPEA (0.049 mL, 0.281 mmol), and di-*tert*-butyl heptane-1,7-diylbis((3-aminopropyl)carbamate) (**7c**) (0.021 g, 0.047 mmol) afforded bis((3a*R*,4*R*,5*R*,7*S*,8*S*,9*R*,9a*S*,12*R*)-8-hydroxy-4,7,9,12-tetramethyl-3-oxo-7-vinyldecahydro-4,9a-propanocyclopenta [8]annulen-5-yl) 7,15-bis(*tert*-butoxycarbonyl)-3,7,15,19-tetraazahenicosanedioate (0.032 g, 58%) as a yellow oil. Following general procedure B, a sub-sample of the protected product (0.016 g, 0.014 mmol) was reacted with TFA in CH_2_Cl_2_ to afford, after chromatography, the tetra-TFA salt **9c** (0.014 g, 72%) as a yellow oil. ${\left[\alpha \right]}_{D}^{21.5}$ = +5 (*c* 0.1, MeOH); R*_f_* (RP-18, 10% *aq* HCl:MeOH 1:3) 0.48; IR (ATR) ν_max_ 2944, 1736, 1676, 1460, 1418, 1200, 1178, 1129, 1051, 1026, 799, 721 cm^−1^; ^1^H NMR (DMSO-*d*_6_, 400 MHz) δ 9.35 (4H, br s, NH_2_-23), 8.74 (4H, br s, NH_2_-27), 6.14 (2H, dd, *J* = 17.8, 11.2 Hz, H-19), 5.63 (2H, d, *J* = 8.3 Hz, H-14), 5.14 (2H, d, *J* = 17.8 Hz, H_2_-20a), 5.06 (2H, d, *J* = 11.3 Hz, H_2_-20b), 4.05 (2H, d, *J* = 17.7 Hz, H_2_-22a), 3.85 (2H, d, *J* = 16.7 Hz, H_2_-22b), 3.45 (2H, d, *J* = 5.5 Hz, H-11), 3.00–2.94 (8H, m, H_2_-24, H_2_-26), 2.90–2.84 (4H, m, H_2_-28), 2.46 (2H, s, H-4), 2.24–1.95 (12H, m, H_2_-2, H-10, H_2_-13a, H_2_-25), 1.69–1.62 (8H, m, H_2_-1b, H_2_-8a, H_2_-29), 1.59–1.50 (2H, m, H-6), 1.40–1.37 (6H, m, H_2_-1a, H_2_-7b, H_2_-13b), 1.38 (6H, s, H_3_-15), 1.32–1.24 (8H, m, H_2_-7a, H_2_-30, H_2_-31), 1.08 (6H, s, H_3_-18), 1.03–1.00 (2H, m, H_2_-8b), 0.83 (6H, d, *J* = 6.8 Hz, H_3_-17), 0.65 (6H, d, *J* = 6.9 Hz, H_3_-16); ^13^C NMR (DMSO-*d*_6_, 100 MHz) δ 217.0 (C-3), 165.6 (C-21), 140.8 (C-19), 115.5 (C-20), 72.4 (C-11), 71.1 (C-14), 57.0 (C-4), 47.4 (C-22), 46.7 (C-28), 44.9 (C-9), 44.2 (C-12), 44.0 (C-24/C-26), 43.8 (C-24/C-26), 43.2 (C-13), 41.5 (C-5), 36.4 (C-10), 36.2 (C-6), 34.0 (C-2), 30.0 (C-8), 28.5 (C-18), 28.1 (C-31), 26.6 (C-7), 25.8 (C-30), 25.3 (C-29), 24.4 (C-1), 22.1 (C-25), 16.2 (C-16), 14.4 (C-15), 11.6 (C-17); (+)-HRESIMS [M + 2H]^+^ *m/z* 483.3684 (calculated for C_57_H_98_N_4_O_8_, 483.3687).

#### 2.2.6. *N*^1^,*N*^1^′-(Octane-1,8-diyl)bis(*N*^3^-(2-(((3a*R*,4*R*,5*R*,7*S*,8*S*,9*R*,9a*S*,12*R*)-8-hydroxy-4,7,9,12-tetramethyl-3-oxo-7-vinyldecahydro-4,9a-propanocyclopenta [8]annulen-5-yl)oxy)-2-oxoethyl)propane-1,3-diaminium) 2,2,2-trifluoroacetate (**9d**)

Following general procedure A, the reaction of pleuromutilin 22-*O*-tosylate (**8**) (0.050 g, 0.094 mmol), KI (0.017 g, 0.103 mmol), DIPEA (0.049 mL, 0.281 mmol), and di-*tert*-butyl octane-1,8-diylbis((3-aminopropyl)carbamate) (**7d**) (0.022 g, 0.047 mmol) afforded bis((3a*R*,4*R*,5*R*,7*S*,8*S*,9*R*,9a*S*,12*R*)-8-hydroxy-4,7,9,12-tetramethyl-3-oxo-7-vinyldecahydro-4,9a-propanocyclopenta [8]annulen-5-yl) 7,16-bis(*tert*-butoxycarbonyl)-3,7,16,20-tetraazadocosanedioate (0.031 g, 56%) as a yellow oil. Following general procedure B, a sub-sample of the protected product (0.015 g, 0.013 mmol) was reacted with TFA in CH_2_Cl_2_ to afford, after chromatography, the tetra-TFA salt **9d** (0.018 g, 99%) as a yellow oil. ${\left[\alpha \right]}_{D}^{20.0}$ = +5 (*c* 0.1, MeOH); R*_f_* (RP-18, 10% *aq* HCl:MeOH 1:3) 0.48; IR (ATR) ν_max_ 2945, 1733, 1670, 1464, 1421, 1202, 1180, 1125, 1025, 798, 720 cm^−1^; ^1^H NMR (DMSO-*d*_6_, 400 MHz) δ 9.33 (4H, br s, NH_2_-23), 8.68 (4H, br s, NH_2_-27), 6.14 (2H, dd, *J* = 17.8, 11.3 Hz, H-19), 5.63 (2H, d, *J* = 8.4 Hz, H-14), 5.14 (2H, dd, *J* = 17.8, 1.7 Hz, H_2_-20a), 5.06 (2H, dd, *J* = 11.2, 1.7 Hz, H_2_-20b), 4.05 (2H, d, *J* = 17.5 Hz, H_2_-22a), 3.85 (2H, d, *J* = 16.9 Hz, H_2_-22b), 3.45 (2H, d, *J* = 5.7 Hz, H-11), 3.00–2.94 (8H, m, H_2_-24, H_2_-26), 2.90–2.85 (4H, m, H_2_-28), 2.45 (2H, s, H-4), 2.22–2.03 (12H, m, H_2_-2, H-10, H_2_-13a), 1.97 (4H, tt, *J* = 7.7, 7.6 Hz, H_2_-25), 1.69–1.62 (4H, m, H_2_-1b, H_2_-8a), 1.56–1.50 (6H, m, H-6, H_2_-29), 1.41–1.37 (4H, m, H_2_-7b, H_2_-13b), 1.38 (6H, s, H_3_-15), 1.30–1.23 (12H, m, H_2_-1a, H_2_-7a, H_2_-30, H_2_-31), 1.08 (6H, s, H_3_-18), 1.03–0.98 (2H, m, H_2_-8b), 0.83 (6H, d, *J* = 7.0 Hz, H_3_-17), 0.65 (6H, d, *J* = 7.0 Hz, H_3_-16); ^13^C NMR (DMSO-*d*_6_, 100 MHz) δ 217.0 (C-3), 165.6 (C-21), 140.8 (C-19), 115.5 (C-20), 72.4 (C-11), 71.1 (C-14), 57.0 (C-4), 47.4 (C-22), 46.6 (C-28), 44.9 (C-9), 44.2 (C-12), 44.0 (C-24/C-26), 43.8 (C-24/C-26), 43.2 (C-13), 41.5 (C-5), 36.5 (C-10), 36.2 (C-6), 34.0 (C-2), 30.0 (C-8), 28.5 (C-18, C-31), 26.6 (C-7), 25.6 (C-30), 25.3 (C-29), 24.4 (C-1), 22.1 (C-25), 16.2 (C-16), 14.4 (C-15), 11.6 (C-17); (+)-HRESIMS [M + 2H]^+^ *m/z* 490.3763 (calculated for C_58_H_100_N_4_O_8_, 490.3765).

#### 2.2.7. *N*^1^,*N*^1^′-(Decane-1,10-diyl)bis(*N*^3^-(2-(((3a*R*,4*R*,5*R*,7*S*,8*S*,9*R*,9a*S*,12*R*)-8-hydroxy-4,7,9,12-tetramethyl-3-oxo-7-vinyldecahydro-4,9a-propanocyclopenta [8]annulen-5-yl)oxy)-2-oxoethyl)propane-1,3-diaminium) 2,2,2-trifluoroacetate (**9e**)

Following general procedure A, the reaction of pleuromutilin 22-*O*-tosylate (**8**) (0.050 g, 0.094 mmol), KI (0.017 g, 0.103 mmol), DIPEA (0.049 mL, 0.281 mmol), and di-*tert*-butyl decane-1,10-diylbis((3-aminopropyl)carbamate) (**7e**) (0.023 g, 0.047 mmol) afforded bis((3a*R*,4*R*,5*R*,7*S*,8*S*,9*R*,9a*S*,12*R*)-8-hydroxy-4,7,9,12-tetramethyl-3-oxo-7-vinyldecahydro-4,9a-propanocyclopenta [8]annulen-5-yl) 7,18-bis(*tert*-butoxycarbonyl)-3,7,18,22-tetraazatetracosanedioate (0.028 g, 49%) as a yellow oil. Following general procedure B, a sub-sample of the protected product (0.014 g, 0.012 mmol) was reacted with TFA in CH_2_Cl_2_ to afford, after chromatography, the tetra-TFA salt **9e** (0.011 g, 65%) as a yellow oil. ${\left[\alpha \right]}_{D}^{19.2}$ = +4 (*c* 0.1, MeOH); R*_f_* (RP-18, 10% *aq* HCl:MeOH 1:3) 0.47; IR (ATR) ν_max_ 2922, 1738, 1669, 1417, 1199, 1129, 1019, 915, 835, 797, 721 cm^−1^; ^1^H NMR (DMSO-*d*_6_, 400 MHz) δ 9.33 (4H, br s, NH_2_-23), 8.68 (4H, br s, NH_2_-27), 6.14 (2H, dd, *J* = 17.8, 11.2 Hz, H-19), 5.63 (2H, d, *J* = 8.3 Hz, H-14), 5.14 (2H, dd, *J* = 17.8, 1.6 Hz, H_2_-20a), 5.06 (2H, dd, *J* = 11.2, 1.5 Hz, H_2_-20b), 4.05 (2H, d, *J* = 16.6 Hz, H_2_-22a), 3.86 (2H, d, *J* = 17.4 Hz, H_2_-22b), 3.45 (2H, d, *J* = 5.5 Hz, H-11), 3.00–2.93 (8H, m, H_2_-24, H_2_-26), 2.90–2.84 (4H, m, H_2_-28), 2.46 (2H, s, H-4), 2.24–2.01 (8H, m, H_2_-2, H-10, H_2_-13a), 1.96 (4H, tt, *J* = 7.5, 7.5 Hz, H_2_-25), 1.69–1.60 (8H, m, H_2_-1b, H_2_-8a, H_2_-29), 1.56–1.50 (2H, m, H-6), 1.40–1.36 (4H, m, H_2_-7b, H_2_-13b), 1.38 (6H, s, H_3_-15), 1.30–1.29 (4H, m, H_2_-1a, H-7a), 1.28–1.24 (12H, m, H_2_-30, H_2_-31, H_2_-32), 1.08 (6H, s, H_3_-18), 1.03–0.99 (2H, m, H_2_-8b), 0.83 (6H, d, *J* = 6.9 Hz, H_3_-17), 0.65 (6H, d, *J* = 7.0 Hz, H_3_-16); ^13^C NMR (DMSO-*d*_6_, 100 MHz) δ 217.0 (C-3), 165.6 (C-21), 140.8 (C-19), 115.5 (C-20), 72.4 (C-11), 71.1 (C-14), 57.0 (C-4), 47.3 (C-22), 46.7 (C-28), 44.9 (C-9), 44.2 (C-12), 44.0 (C-24/C-26), 43.8 (C-24/C-26), 43.2 (C-13), 41.5 (C-5), 36.4 (C-10), 36.2 (C-6), 34.0 (C-2), 30.0 (C-8), 28.7 (C-32), 28.54 (C-18/C-31), 28.51 (C-18/C-31), 26.6 (C-7), 25.9 (C-30), 25.4 (C-29), 24.4 (C-1), 22.1 (C-25), 16.2 (C-16), 14.4 (C-15), 11.6 (C-17); (+)-HRESIMS [M + 2H]^+^ *m/z* 504.3922 (calculated for C_60_H_104_N_4_O_8_, 504.3922).

#### 2.2.8. *N*^1^,*N*^1^′-(Dodecane-1,12-diyl)bis(*N*^3^-(2-(((3a*R*,4*R*,5*R*,7*S*,8*S*,9*R*,9a*S*,12*R*)-8-hydroxy-4,7,9,12-tetramethyl-3-oxo-7-vinyldecahydro-4,9a-propanocyclopenta [8]annulen-5-yl)oxy)-2-oxoethyl)propane-1,3-diaminium) 2,2,2-trifluoroacetate (**9f**)

Following general procedure A, the reaction of pleuromutilin 22-*O*-tosylate (**8**) (0.050 g, 0.094 mmol), KI (0.017 g, 0.103 mmol), DIPEA (0.049 mL, 0.281 mmol), and di-*tert*-butyl dodecane-1,12-diylbis((3-aminopropyl)carbamate) (**7f**) (0.024 g, 0.047 mmol) afforded bis((3a*R*,4*R*,5*R*,7*S*,8*S*,9*R*,9a*S*,12*R*)-8-hydroxy-4,7,9,12-tetramethyl-3-oxo-7-vinyldecahydro-4,9a-propanocyclopenta [8]annulen-5-yl) 7,20-bis(*tert*-butoxycarbonyl)-3,7,20,24-tetraazahexacosanedioate (0.023 g, 40%) as a yellow oil. Following general procedure B, a sub-sample of the protected product (0.014 g, 0.011 mmol) was reacted with TFA in CH_2_Cl_2_ to afford, after chromatography, the tetra-TFA salt **9f** (0.008 g, 47%) as a yellow oil. ${\left[\alpha \right]}_{D}^{19.7}$ = +5 (*c* 0.1, MeOH); R*_f_* (RP-18, 10% *aq* HCl:MeOH 1:3) 0.40; IR (ATR) ν_max_ 2930, 1735, 1671, 1499, 1417, 1199, 1175, 1125, 1018, 915, 834, 797, 721 cm^−1^; ^1^H NMR (DMSO-*d*_6_, 500 MHz) δ 9.32 (4H, br s, NH_2_-23), 8.62 (4H, br s, NH_2_-27), 6.14 (2H, dd, *J* = 17.7, 11.2 Hz, H-19), 5.63 (2H, d, *J* = 8.3 Hz, H-14), 5.14 (2H, d, *J* = 17.8 Hz, H_2_-20a), 5.06 (2H, d, *J* = 11.2 Hz, H_2_-20b), 4.03 (2H, d, *J* = 17.0 Hz, H_2_-22a), 3.84 (2H, d, *J* = 17.1 Hz, H_2_-22b), 3.45 (2H, obscured by H_2_O, H-11), 2.96 (8H, t, *J* = 7.2 Hz, H_2_-24, H_2_-26), 2.87 (4H, t, *J* = 7.8 Hz, H_2_-28), 2.46 (2H, br s, H-4), 2.23–2.01 (8H, m, H_2_-2, H-10, H_2_-13a), 1.95 (4H, tt, *J* = 7.3, 7.2 Hz, H_2_-25), 1.69–1.51 (10H, m, H_2_-1b, H-6, H_2_-8a, H_2_-29), 1.39–1.34 (4H, m, H_2_-7b, H_2_-13b), 1.38 (6H, s, H_3_-15), 1.31–1.28 (4H, m, H_2_-1a, H_2_-7a), 1.28–1.23 (16H, m, H_2_-30, H_2_-31, H_2_-32, H_2_-33), 1.08 (6H, s, H_3_-18), 1.03–1.00 (2H, m, H_2_-8b), 0.83 (6H, d, *J* = 6.8 Hz, H_3_-17), 0.65 (6H, d, *J* = 7.0 Hz, H_3_-16); ^13^C NMR (DMSO-*d*_6_, 125 MHz) δ 217.0 (C-3), 165.8 (C-21), 140.8 (C-19), 115.5 (C-20), 72.4 (C-11), 71.0 (C-14), 57.0 (C-4), 47.4 (C-22), 46.7 (C-28), 44.9 (C-9), 44.2 (C-12), 44.0 (C-24/C-26), 43.9 (C-24/C-26), 43.2 (C-13), 41.5 (C-5), 36.5 (C-10), 36.2 (C-6), 34.0 (C-2), 30.0 (C-8), 29.0 (C-33), 28.9 (C-32), 28.6 (C-31), 28.5 (C-18), 26.6 (C-7), 25.9 (C-30), 25.4 (C-29), 24.4 (C-1), 22.2 (C-25), 16.2 (C-16), 14.4 (C-15), 11.6 (C-17); (+)-HRESIMS [M + 2H]^+^ *m/z* 518.4083 (calculated for C_62_H_108_N_4_O_8_, 518.4078).

### 2.3. Antimicrobial Assays

The susceptibility of bacterial strains *S. aureus* (ATCC 25923), *E. coli* (ATCC 25922), and *P. aeruginosa* (ATCC 27853 or PAO1) to antibiotics and compounds was determined using reported protocols [26]. Additional antimicrobial evaluation against MRSA (ATCC 43300), *Klebsiella pneumoniae* (ATCC 700603), *A. baumannii* (ATCC 19606), *Candida albicans* (ATCC 90028), and *Cryptococcus neoformans* (ATCC 208821) was undertaken at the Community for Open Antimicrobial Drug Discovery at The University of Queensland (Australia) according to their standard protocols as reported previously [26,27]. (See Supplementary File.)

### 2.4. Determination of the MICs of Antibiotics in the Presence of Synergizing Compounds

Antibiotic restoring enhancer concentrations were determined using reported protocols (see Supplementary File) [26].

### 2.5. Nitrocefin Assay

Nitrocefin assays were conducted using reported protocols (see Supplementary File) [28].

### 2.6. Cytotoxicity Assays

Cytotoxicity assays were conducted using reported protocols (see Supplementary File) [26,27].

### 2.7. Hemolytic Assays

Hemolysis assays were conducted using reported protocols (see Supplementary File) [26,27].

### 2.8. Real-Time Growth Curves

Real-time growth curves were determined using reported protocols (see Supplementary File) [26].

### 2.9. ATP Efflux Assay

ATP efflux assays were conducted using reported protocols (see Supplementary File) [28].

## 3. Results and Discussion

### 3.1. Synthesis of Tiamulin (**2**)

**Scheme 1:**
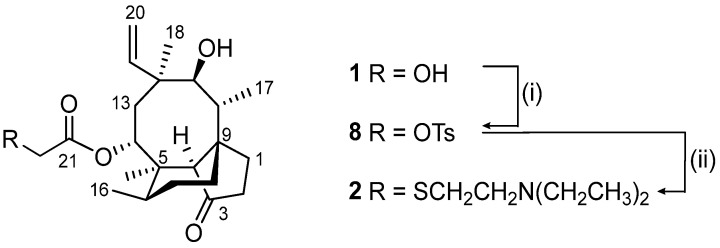
Synthesis of pleuromutilin *O*-22-tosylate (**8**) and tiamulin (**2**).

Functionalization at C-22 of pleuromutilin (**1**) has been extensively explored, with derivatives prepared via nucleophilic substitution of sulphonate esters. Tosylation at *O*-22 was achieved using a modified procedure by Zhang et al. [29], whereby pleuromutilin (**1**) in CH_2_Cl_2_ was reacted with 4-toluenesulfonylchloride and DMAP for 4 h to afford **8** in 57% yield (Scheme 1).

Reagents and conditions: (i) 4-Toluenesulfonylchloride (1.2 equiv), DMAP (3 equiv.), CH_2_Cl_2_, 4 h, 70 °C, N_2_ (57%); (ii) KI (2 equiv.), MeCN, 70 °C, 30 min, N_2_, then 2-(diethylamino)ethane-1-thiol hydrochloride (1.1 equiv.), and DIPEA (6 equiv.), 70 °C, 2 h, N_2_ (47%).

Tiamulin (**2**) was selected to be used as a positive control in biological assays and was prepared by a one pot, two-step sequence, whereby 22-*O*-tosylate **8** was preincubated with 2.0 equivalents of KI to give the 22-iodo derivative which was then reacted with 1.1 equivalents of 2-(diethylamino)ethane-1-thiol HCl salt in the presence of excess DIPEA to afford (**2**) in 47% yield (Scheme 1) (Figure S1).

### 3.2. Synthesis of Pleuromutilin–Polyamine Conjugates

**Figure 2:**
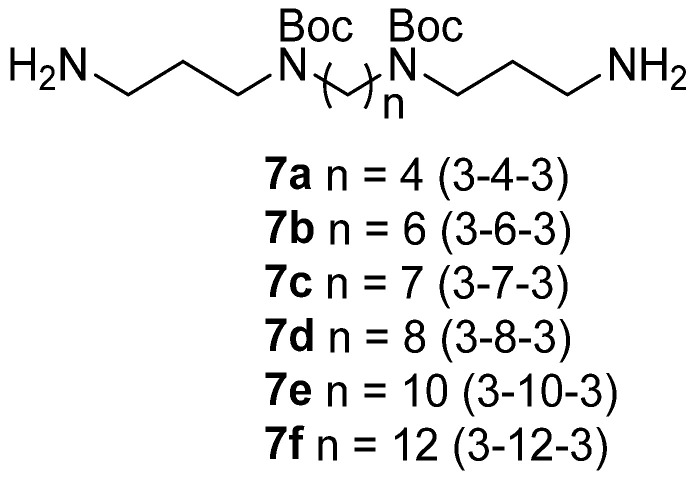
Boc-protected polyamines **7a**–**f**.

The target set of conjugates required for the synthesis of Boc-protected polyamine scaffolds **7a**–**f** (Figure 2), which were prepared according to protocols reported in the literature [22,23,24]. The polyamines (PA) chosen covered a range of overall lengths, from spermine (PA-3-4-3) through to the longer chain length PA-3-12-3. The set was chosen to allow the exploration of chain length, lipophilicity, and positioning of positive charges on antimicrobial and cytotoxicity/hemolytic properties.

**Scheme 2:**
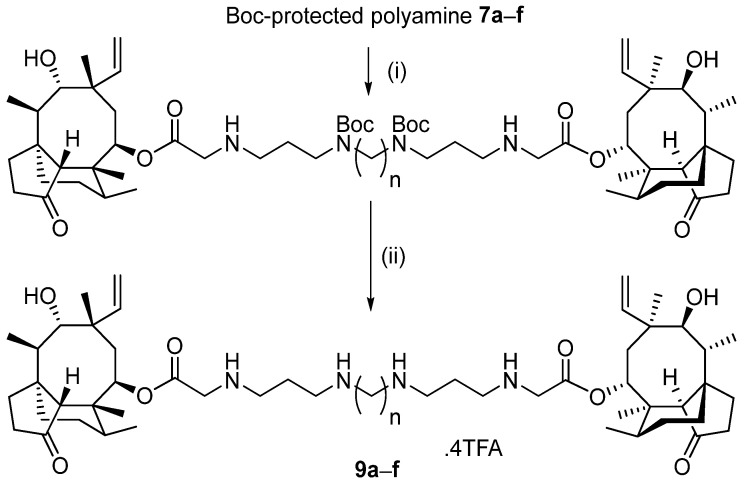
General method for the synthesis of target pleuromutilin–polyamine conjugates (**9a**–**f**).

The target pleuromutilin–polyamine conjugates were prepared using the same nucleophilic displacement of the iodo-activated pleuromutilin methodology used to prepare tiamulin (**2**). Thus, activation of pleuromutilin 22-*O*-tosylate (**8**) with KI, followed by reaction with Boc-protected polyamines **7a**–**f** in MeCN with DIPEA, afforded Boc-protected intermediates that were then deprotected with 2,2,2-trifluoroacetic acid (TFA) to afford target compounds **9a**–**f** as their tetra-TFA salts (Scheme 2) (Figures S2–S7).

Regents and conditions: (i) Pleuromutilin *O*-22-tosylate (**8**) (2.0 equiv.), KI (2.2 equiv.), MeCN, 70 °C, 1.5 h, N_2_, then Boc-protected polyamine **7a**–**f** (1.0 equiv.), and DIPEA (6 equiv.), 70 °C, 2.5 h, N_2_ (40–72%); (ii) TFA (0.2 mL), CH_2_Cl_2_ (2 mL), r.t., 2 h (47–99%).

### 3.3. Antimicrobial Activities

The antimicrobial activities of pleuromutilin (**1**), tiamulin (**2**), and pleuromutilin–polyamine conjugates **9a**–**f** were determined as the minimum inhibitory concentration (MIC) against a panel of Gram-positive (*S. aureus* and MRSA) and Gram-negative (*E. coli*, *P. aeruginosa, K. pneumoniae,* and *A. baumannii*) bacterial strains and two fungal strains (*C. albicans* and *C. neoformans*) (Table 1). Tiamulin (**2**) demonstrated potent activity against both *S. aureus* and MRSA with an MIC of 3.125 and ≤0.51 μM, respectively, while exhibiting no activity against any of the Gram-negative bacteria or fungi. Intriguingly, all of the conjugates exhibited moderate to good growth inhibition of *S. aureus* and the Gram-negative bacterium, *E. coli,* with the conjugates **9b**–**f** also demonstrating strong growth inhibition of MRSA. Antifungal activity was observed for all conjugates against *C*. *neoformans,* with MIC’s ranging from ≤0.17 to 0.72 μM, while against *C*. *albicans*, activity was only observed for the longer chained PA-3-10-3 **9e** (MIC 10.9 μM) and PA-3-12-3 **9f** (MIC 21.5 μM) conjugates. Meanwhile, the PA-3-12-3 conjugate **9f** was the most active, exhibiting broad spectrum activity against all tested strains, notably against *S. aureus*, MRSA, *E. coli*, *A. baumannii,* and *C. neoformans*.

### 3.4. Cytotoxic and Hemolytic Activities

Tiamulin (**2**) and pleuromutilin–polyamine conjugates **9a**–**f** were evaluated for cytotoxicity towards HEK293 cells (human kidney epithelial cell line), reported as the concentration of compound at 50% cytotoxicity (IC_50_), and for hemolytic activity against human red blood cells, reported as the concentration of compound at 10% hemolytic activity (HC_10_) (Table 2). The only conjugate to exhibit either of these properties was the longest PA-3-12-3 variant (**9f**), which was both cytotoxic (IC_50_ 8.3 µM) and hemolytic (HC_10_ 13.2 µM).

When taken together, the combination of intrinsic antimicrobial activities and cytotoxicity/hemolytic activities identified the PA-3-10-3 conjugate **9e** as being of particular interest. Of note, in addition to the strong activity towards Gram-positive bacteria, the conjugate also exhibited activity towards the Gram-negative bacterium *E. coli*.

### 3.5. Real-Time Growth Inhibition Assay

**Figure 3:**
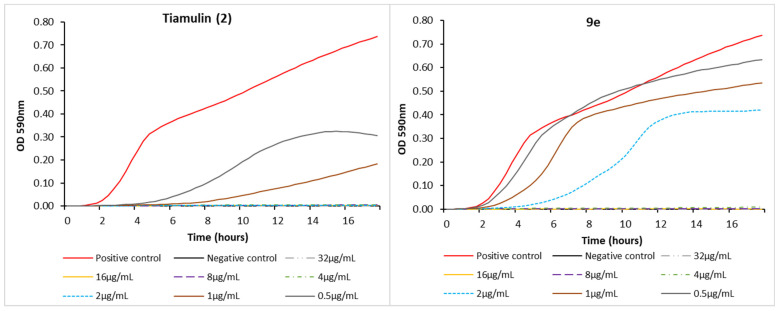
Bacterial growth inhibition exhibited by **2** (**left**) and **9e** (**right**) against *S. aureus* ATCC 25923 at different concentrations. Positive control was bacteria only and negative control was media only.

To investigate the kinetics of the antibacterial activity exhibited by **9e** and tiamulin (**2**), real-time growth inhibition curves were determined against *S. aureus* ATCC 25922 and *P. aeruginosa* PAO1 via measurement of optical density at 490 nm during an 18 h culturing period. Although appearing to be qualitatively different, both compounds inhibited the growth of *S. aureus* (Figure 3), with the 18 h time point values in close agreement with the MIC values obtained for the two compounds (Table 1) using classical microdilution techniques. Further investigation is required to determine the factors leading to the differences observed in the sub-MIC growth curves for the two compounds. No such inhibition was observed against *P. aeruginosa* (Figure S8).

### 3.6. Antibiotic Enhancement Activities

Tiamulin (**2**) and polyamine conjugates **9a**–**f** were also evaluated for their ability to enhance the antibiotic action of doxycycline towards *P. aeruginosa* ATCC 27853 and of erythromycin towards *E. coli* ATCC 25922. For the doxycycline assay, the antibiotic was present at a concentration of 4.5 μM (2 μg/mL), 20 times below the MIC value of 90 μM (40 μg/mL). Amongst the test compounds, only modest levels of enhancement were detected, with 4-fold increases in activity observed for **9a**, **9b,** and **9f**, while the remaining compounds, including tiamulin (**2**), were essentially unable to enhance the antibiotic action of doxycycline (Table 3). For the erythromycin assay, the antibiotic was present at a concentration of 2.7 μM (2 μg/mL), well below the MIC value of 174 μM (128 μg/mL). Insignificant levels of enhancement of the action of the lipophilic antibiotic erythromycin was detected towards *E. coli*, though of note is the observance of improved activity for three of the conjugates (**9a**, **9c**, and **9d**) in comparison to tiamulin (**2**).

### 3.7. Membrane Perturbation Activities

Perturbation or disruption of the bacterial membrane is a validated mechanism of action of particular classes of antibiotics, including the polymyxin family of lipopeptides. In addition to being lethal to bacteria, this mechanism of membrane perturbation can also be used to enhance the action of other antibiotics, whereby a sub-MIC dose of the disruptor can facilitate the entry of the antibiotics into the microorganism [30]. Membrane perturbation appears to be one mechanism of both antibiotic activity and antibiotic enhancement activity exhibited by α,ω-disubstituted polyamines, being well documented in a number of studies [21].

**Figure 4:**
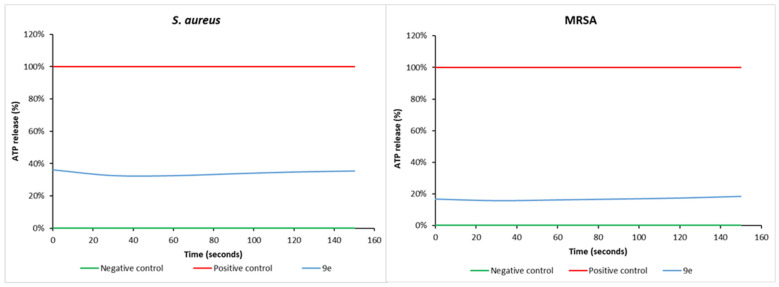
ATP release in *S. aureus* ATCC 25923 (**left**) and MRSA (**right**) exhibited by polyamine conjugate **9e** as determined using ATP efflux assay. Squalamine (100 µg/mL) was the positive control and water was the negative control. Compounds were tested at a final concentration of 100 µg/mL, and results are reported as percentage (%) relative to positive control.

The mechanism of antibiotic action of **9e** towards the Gram-positive bacterium *Staphylococcus aureus* was attributed to its ability to disrupt the bacterial membrane and cause the release of intracellular ATP. The test organisms, being *S. aureus* ATCC 25923 and MRSA, were briefly exposed to **9e** at a single dose of 100 µg/mL, with the leakage of ATP determined by the use of a bioluminescence assay (Figure 4). While considered active in the assay, the level of disruption induced by exposure to **9e** was significantly less than that induced by the positive control squalamine. Nevertheless, a 40% level of ATP release is detrimental to the survival of the bacteria. After 3 min, it will be clear that the bacteria will be killed.

**Figure 5:**
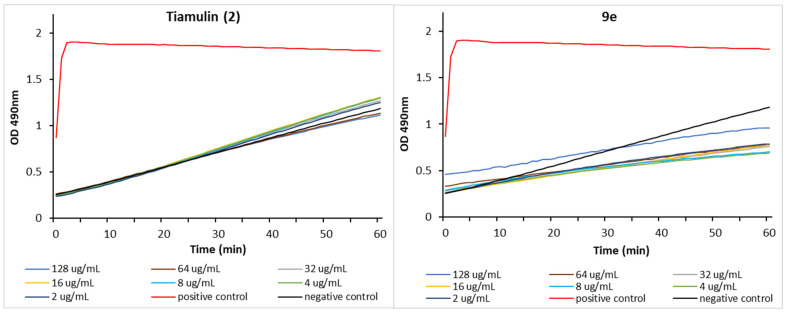
The ability of tiamulin (**2**) (**left**) and polyamine conjugate **9e** (**right**) to act as membrane disruptors in *P. aeruginosa* PAO1, as determined using a nitrocefin hydrolysis assay. Polymixin B (PMB) was the positive control (98.3 μM, 128 μg/mL), and the negative control was bacteria with nitrocefin.

We also examined the ability of **9e** to act as membrane disruptor of the Gram-negative bacteria *P. aeruginosa* PAO1 (Figure 5), using a nitrocefin colorimetric assay. This assay makes use of a chromogenic cephalosporin derivative, which in the presence of an outer membrane disruptor gains entry to the periplasm, where upon the action of β-lactamases leads to substrate hydrolysis with a detectable color change from yellow to red. While the positive control, polymyxin B, demonstrated potent ability to perturb the outer membrane of *P. aeruginosa* PAO1, polyamine conjugate **9e** was inactive at all test doses. Tiamulin (**2**) was also evaluated in the same assay, also failing to exhibit any detectable membrane perturbation.

## 4. Conclusions

Six pleuromutilin–polyamine conjugates **9a**–**f** were successfully synthesized from pleuromutilin-22-OTs (**8**) and Boc-protected polyamines **7a**–**f**, in addition to the veterinary drug tiamulin (**2**). These compounds primarily inhibited the growth of Gram-positive bacteria, with limited effects on Gram-negative bacteria and two fungal strains. Notably, all polyamine conjugates exhibited anti-*E. coli* and antifungal activity against *C. neoformans*, unlike pleuromutilin (**1**) or tiamulin (**2**). Cytotoxicity and hemolysis assays showed that all compounds, except the longest polyamine conjugate, **9f**, were non-toxic. Moreover, when combined with doxycycline against *P. aeruginosa*, three conjugates (**9a**, **9b**, and **9f**) exhibited better antibiotic potentiation than tiamulin (**2**). Preliminary investigations suggest that while **9e** exhibits intrinsic Gram-positive antibacterial by a mechanism related to membrane perturbation, the ability of the compound class to enhance the action of doxycycline towards Gram-negative bacteria does not appear to be linked to outer membrane disruption. Further studies will be required to determine their precise mechanism.

## Figures and Tables

**Table 1:** Antimicrobial (MIC, μM) activities of pleuromutilin (**1**), tiamulin (**2**), and conjugates **9a**–**f**.

Compound	S.a. ^a^	MRSA ^b^	E.c. ^c^	P.a. ^d^	K.p. ^e^	A.b. ^f^	C.a. ^g^	C.n. ^h^
**1**	3.125	n.t. ^i^	400	800	n.t.	n.t.	n.t.	n.t.
**2**	3.125	≤0.51	200	800	>64.8	>64.8	>64.8	>64.8
**9a**	18.1	23.2	18.1	72.5	>23.2	>23.2	>23.2	0.72
**9b**	17.8	≤0.18	8.88	142	>22.7	>22.7	>22.7	≤0.18
**9c**	17.6	≤0.18	35.2	>141	>22.5	>22.5	>22.5	≤0.18
**9d**	34.8	5.57	70	>139	>22.3	>22.3	>22.3	≤0.17
**9e**	4.27	≤0.17	4.27	>88	>21.9	>21.9	10.9	≤0.17
**9f**	2.10	≤0.17	4.19	33.5	21.5	2.7	21.5	≤0.17

^a^ *S. aureus* ATCC 25923, streptomycin (MIC 21.5 μM) and chloramphenicol (MIC 1.5–3 μM) used as positive controls and values presented as the mean (n = 3); ^b^ MRSA ATCC 43300, vancomycin (MIC 0.7 μM) used as a positive control and values presented as the mean (n = 2); ^c^ *E. coli* ATCC 25922, streptomycin (MIC 21.5 μM) and colistin (MIC 2 μM) used as positive controls and values presented as the mean (n = 3); ^d^ *P. aeruginosa* PAO1, streptomycin (MIC 21.5 μM) and colistin (MIC 1 μM) used as positive controls and values presented as the mean (n = 3); ^e^ *K. pneumoniae* ATCC 700603, colistin (MIC 0.2 µM) used as a positive control and values presented as the mean (n = 2); ^f^ *A. baumannii* ATCC 19606, colistin (MIC 0.2 µM) used as a positive control and values presented as the mean (n = 2); ^g^ *C. albicans* ATCC 90028, fluconazole (MIC 0.4 μM) used as a positive control and values presented as the mean (n = 2); ^h^ *C. neoformans* ATCC 208821, fluconazole (MIC 26 µM) used as a positive control and values presented as the mean (n = 2); ^i^ Not tested.

**Table 2:** Cytotoxicity (IC_50_, μM) and hemolytic (HC_10_, μM) activities of tiamulin (**2**) and conjugates **9a**–**f**.

Compound	Cytotoxicity ^a^	Hemolysis ^b^
**2**	>64.8	>64.8
**9a**	>23.2	>23.2
**9b**	>22.7	>22.7
**9c**	>22.5	>22.5
**9d**	>22.3	>22.3
**9e**	>21.9	>21.9
**9f**	8.3	13.2

^a^ Concentration of compound at 50% cytotoxicity on HEK293 human embryonic kidney cells with tamoxifen as the positive control (IC_50_ 24 µM) and values presented as the mean (n = 2); ^b^ Concentration of compound at 10% hemolytic activity on human red blood cells with melittin as the positive control (HC_10_ 0.95 µM) and values presented as the mean (n = 2).

**Table 3:** Antibiotic enhancement activity (MIC, µM) of tiamulin (**2**) and conjugates **9a**–**f**.

Compound	Dox/P.a. ^a^	Erythro/E.c. ^b^
**2**	>405 (2)	>405 (0.5)
**9a**	18.1 (4)	9.06 (2)
**9b**	35.5 (4)	8.88 (1)
**9c**	141 (1)	17.6 (2)
**9d**	139 (1)	34.8 (2)
**9e**	34.2 (2)	8.54 (0.5)
**9f**	8.38 (4)	8.38 (0.5)

^a^ Concentration (µM) required to restore doxycycline activity at 4.5 µM against *P. aeruginosa* ATCC 27853. Fold change shown in parentheses is the ratio between the intrinsic MIC of the test compound and the combination MIC; ^b^ Concentration (µM) required to restore erythromycin activity at 2.7 µM against *E. coli* ATCC 25922. Fold change shown in parentheses is the ratio between the intrinsic MIC of the test compound and the combination MIC.

## Data Availability

Data are contained within the article or Supplementary Materials.

## Supplementary Materials

The following supporting information can be downloaded at: https://www.mdpi.com/article/10.3390/microorganisms11112791/s1, Figure S1: ^1^H (CDCl_3_, 400 MHz) and ^13^C (CDCl_3_, 100 MHz) NMR spectra for **2**; Figure S2: ^1^H (DMSO-*d*_6_, 400 MHz) and ^13^C (DMSO-*d*_6_, 100 MHz) NMR spectra for **9a**; Figure S3: ^1^H (DMSO-*d*_6_, 400 MHz) and ^13^C (DMSO-*d*_6_, 100 MHz) NMR spectra for **9b**; Figure S4: ^1^H (DMSO-*d*_6_, 400 MHz) and ^13^C (DMSO-*d*_6_, 100 MHz) NMR spectra for **9c**; Figure S5: ^1^H (DMSO-*d*_6_, 400 MHz) and ^13^C (DMSO-*d*_6_, 100 MHz) NMR spectra for **9d**; Figure S6: ^1^H (DMSO-*d*_6_, 400 MHz) and ^13^C (DMSO-*d*_6_, 100 MHz) NMR spectra for **9e**; Figure S7: ^1^H (DMSO-*d*_6_, 500 MHz) and ^13^C (DMSO-*d*_6_, 125 MHz) NMR spectra for **9f**; Figure S8: Bacterial growth inhibition exhibited by **2** (left) and **9e** (right) against *P. aeruginosa* PAO1 at different concentrations. Positive control was bacteria only and negative control was media only. The Supplementary File contains full protocols for all bioassays conducted.
