# Supplementary material for: Preliminary SAR of Novel Pleuromutilin–Polyamine Conjugates
Source: Microorganisms. 2023 Nov 17;11(11):2791. doi: 10.3390/microorganisms11112791 (PMC10673369; doi:10.3390/microorganisms11112791)
Supplement: Supplementary file 1 [file microorganisms-11-02791-s001.zip › microorganisms-2610679-supplemetary-Figures.pdf]

Supplementary Materials

Preliminary SAR of Novel Pleuromutilin-Polyamine Conjugates

Kenneth Sue [1], Melissa M. Cadelis [1,2], Kerrin Hainsworth [1], Florent Rouvier [3], Marie-Lise Bourguet-Kondracki [4], Jean Michel Brunel [3] and Brent R. Copp [1,*]

[1] School of Chemical Sciences, The University of Auckland, Private Bag 92019, Auckland 1142, New Zealand
[2] School of Medical Sciences, The University of Auckland, Private Bag 92019, Auckland 1142, New Zealand
[3] Membranes et Cibles Thérapeutiques, INSERM, Aix-Marseille Universite, 27 bd Jean Moulin, 13385 Marseille, France
[4] Laboratoire Molécules de Communication et Adaptation des Micro-organismes, UMR 7245 CNRS, Muséum National d'Histoire Naturelle, 57 rue Cuvier (C.P. 54), 75005 Paris, France
* Correspondence: b.copp@auckland.ac.nz

Contents

Figure S1 ^{1}H ($CDCl_3$, 400 MHz) and ^{13}C ($CDCl_3$, 100 MHz) NMR spectra for **2**. S2

Figure S2 ^{1}H (DMSO-d_6, 400 MHz) and ^{13}C (DMSO-d_6, 100 MHz) NMR spectra for **9a**. S3

Figure S3 ^{1}H (DMSO-d_6, 400 MHz) and ^{13}C (DMSO-d_6, 100 MHz) NMR spectra for **9b**. S4

Figure S4 ^{1}H (DMSO-d_6, 400 MHz) and ^{13}C (DMSO-d_6, 100 MHz) NMR spectra for **9c**. S5

Figure S5 ^{1}H (DMSO-d_6, 400 MHz) and ^{13}C (DMSO-d_6, 100 MHz) NMR spectra for **9d**. S6

Figure S6 ^{1}H (DMSO-d_6, 400 MHz) and ^{13}C (DMSO-d_6, 100 MHz) NMR spectra for **9e**. S7

Figure S7 ^{1}H (DMSO-d_6, 500 MHz) and ^{13}C (DMSO-d_6, 125 MHz) NMR spectra for **9f**. S8

Figure S8 Bacterial growth inhibition exhibited by **2** (left) and **9e** (right) against *P. aeruginosa* PAO1 at different concentrations. Positive control was bacteria only and negative control was media only. S9

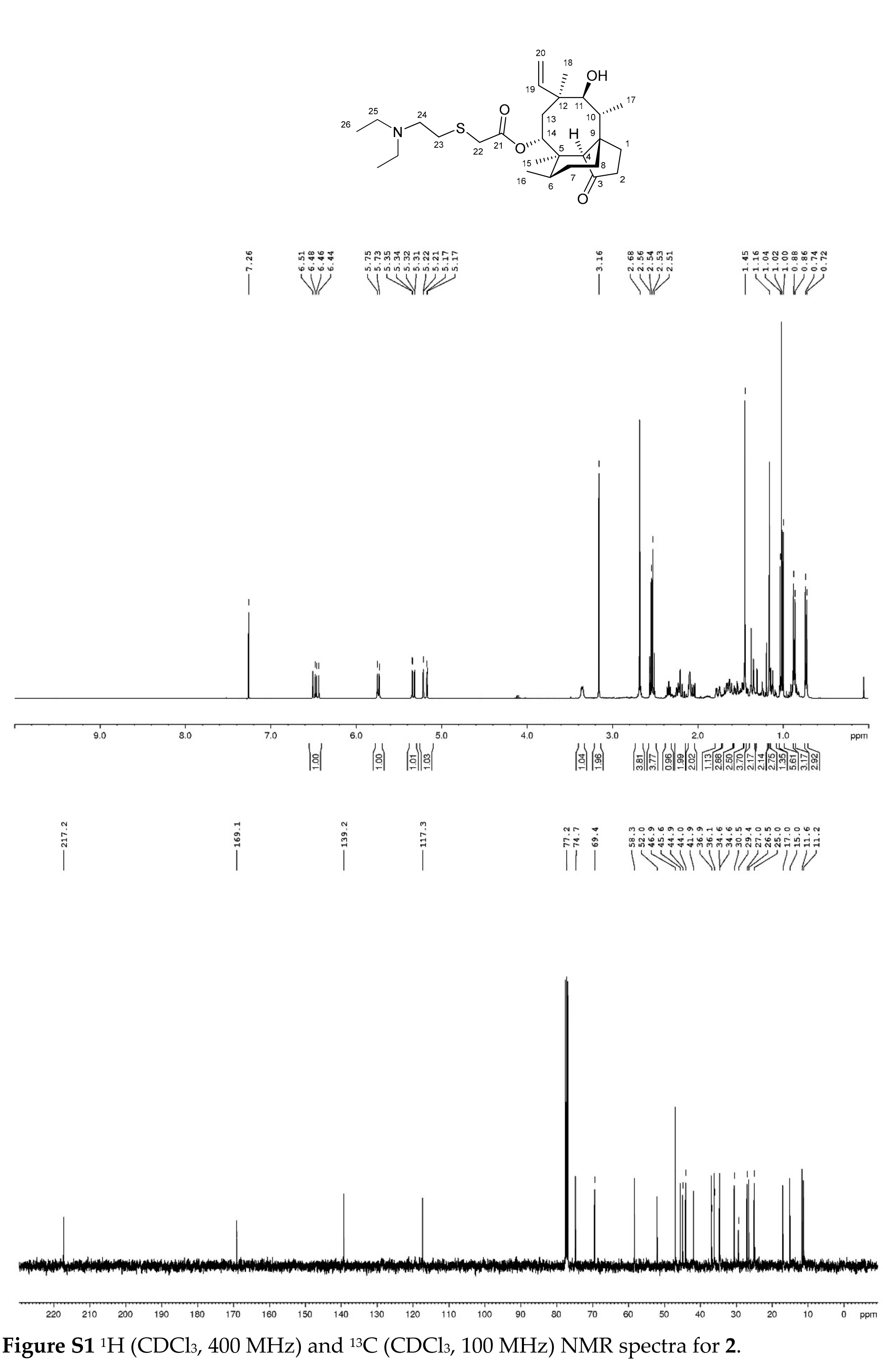

Figure S1 ^{1}H ($CDCl_3$, 400 MHz) and ^{13}C ($CDCl_3$, 100 MHz) NMR spectra for **2**.

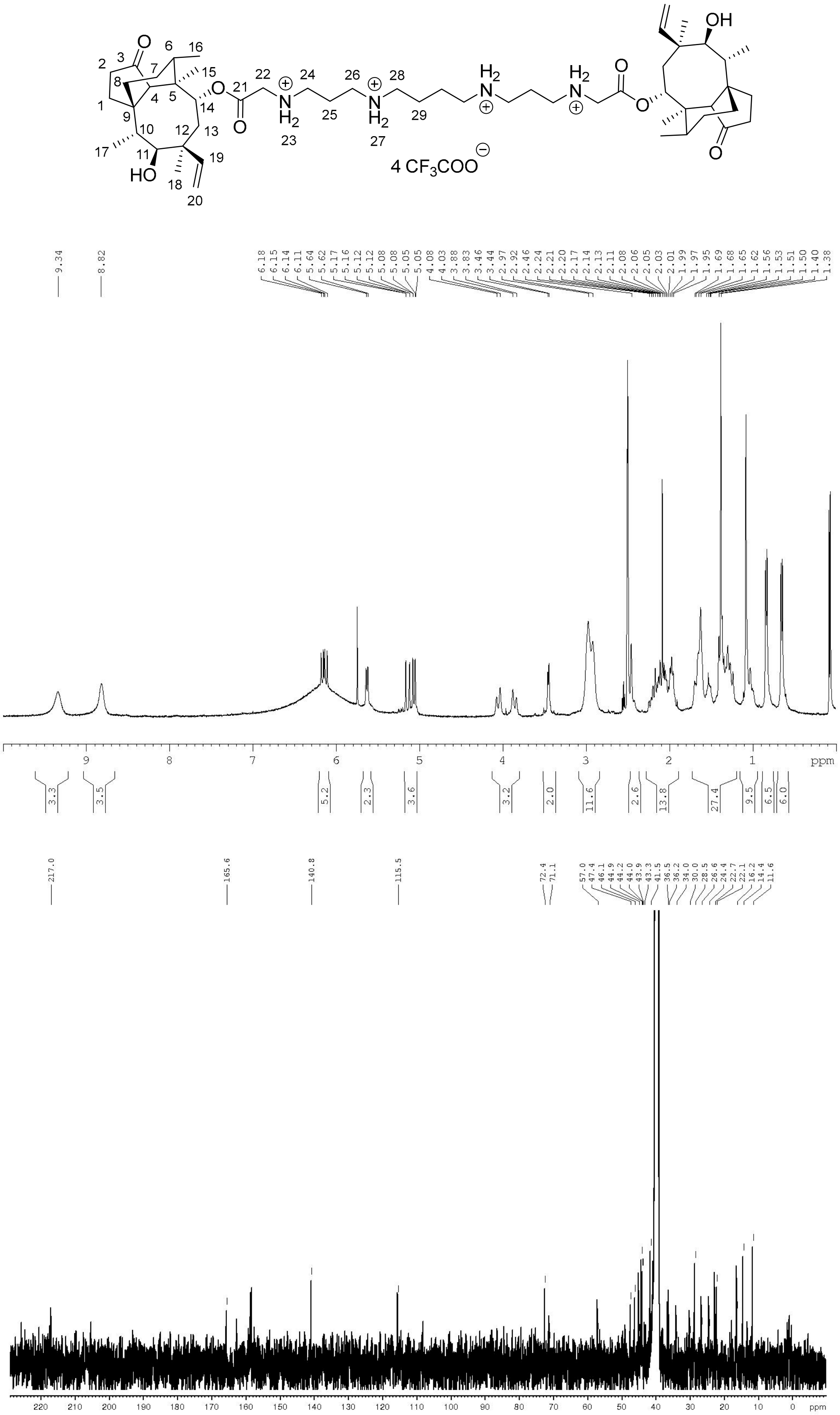

Figure S2 ^{1}H (DMSO-d_6, 400 MHz) and ^{13}C (DMSO-d_6, 100 MHz) NMR spectra for **9a**.

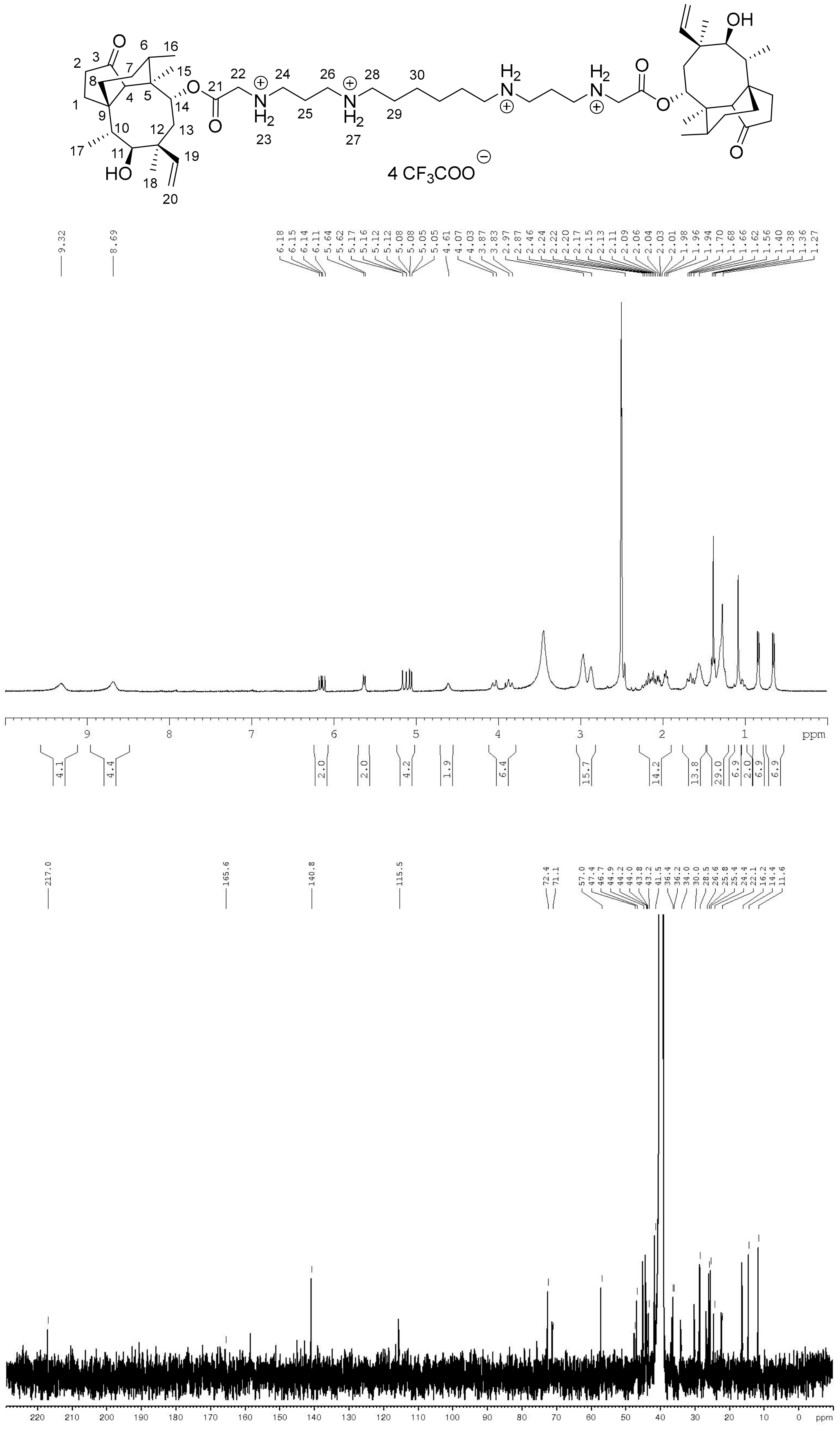

Figure S3 ^{1}H (DMSO-d_6, 400 MHz) and ^{13}C (DMSO-d_6, 100 MHz) NMR spectra for **9b**.

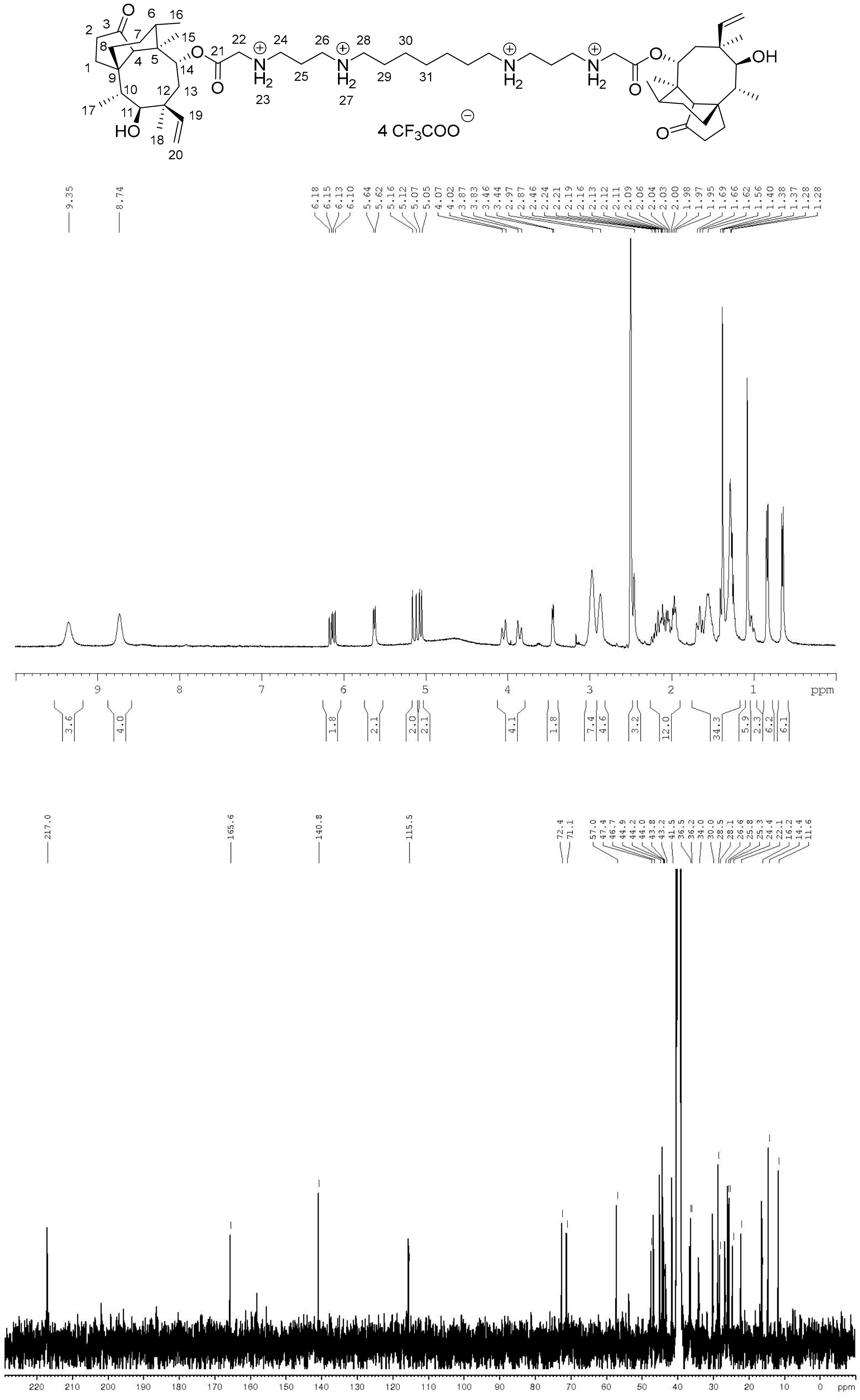

Figure S4 ^{1}H (DMSO-d_6, 400 MHz) and ^{13}C (DMSO-d_6, 100 MHz) NMR spectra for **9c**.

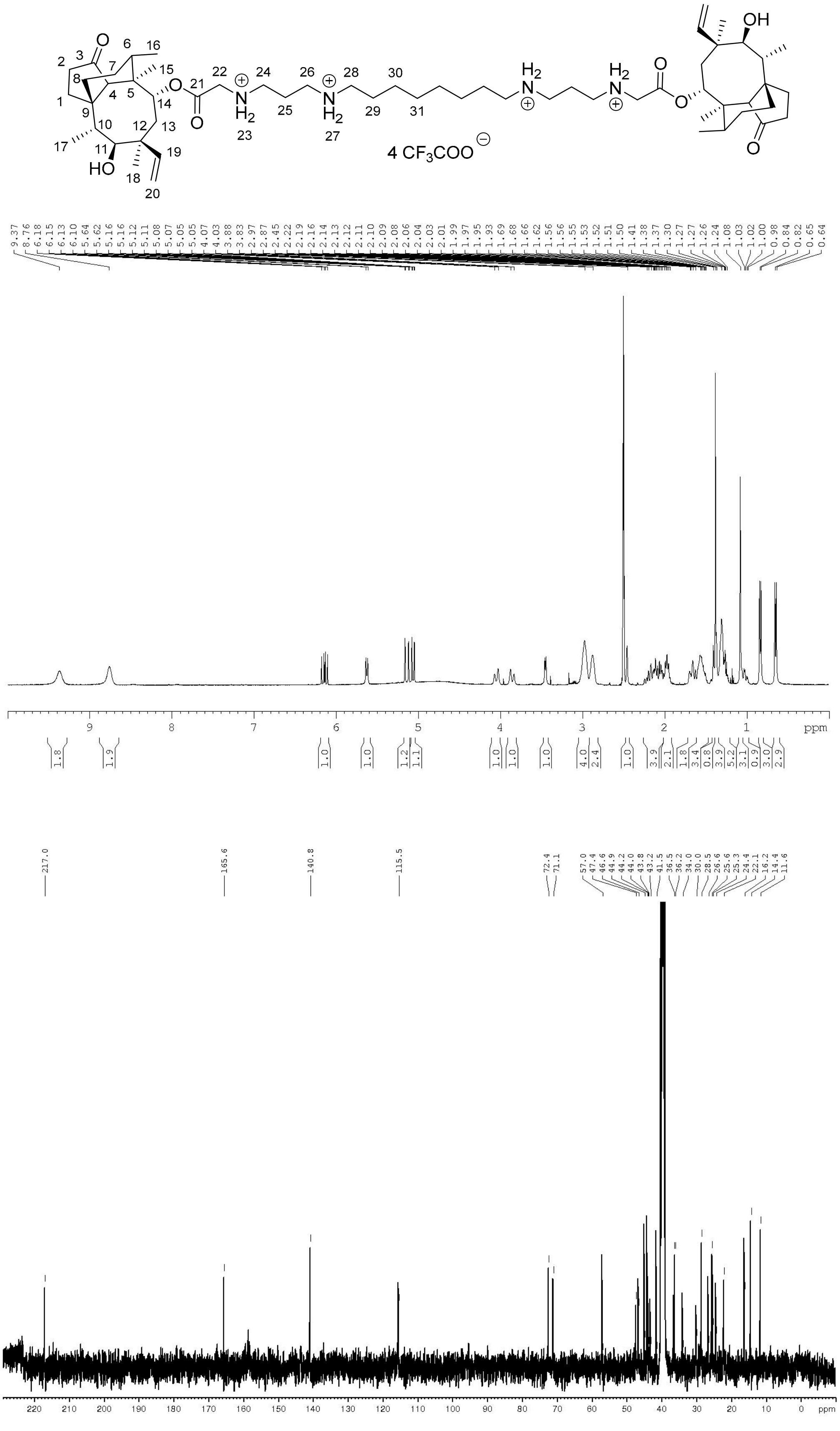

Figure S5 ^{1}H (DMSO-*d*$_6$, 400 MHz) and ^{13}C (DMSO-*d*$_6$, 100 MHz) NMR spectra for **9d**.

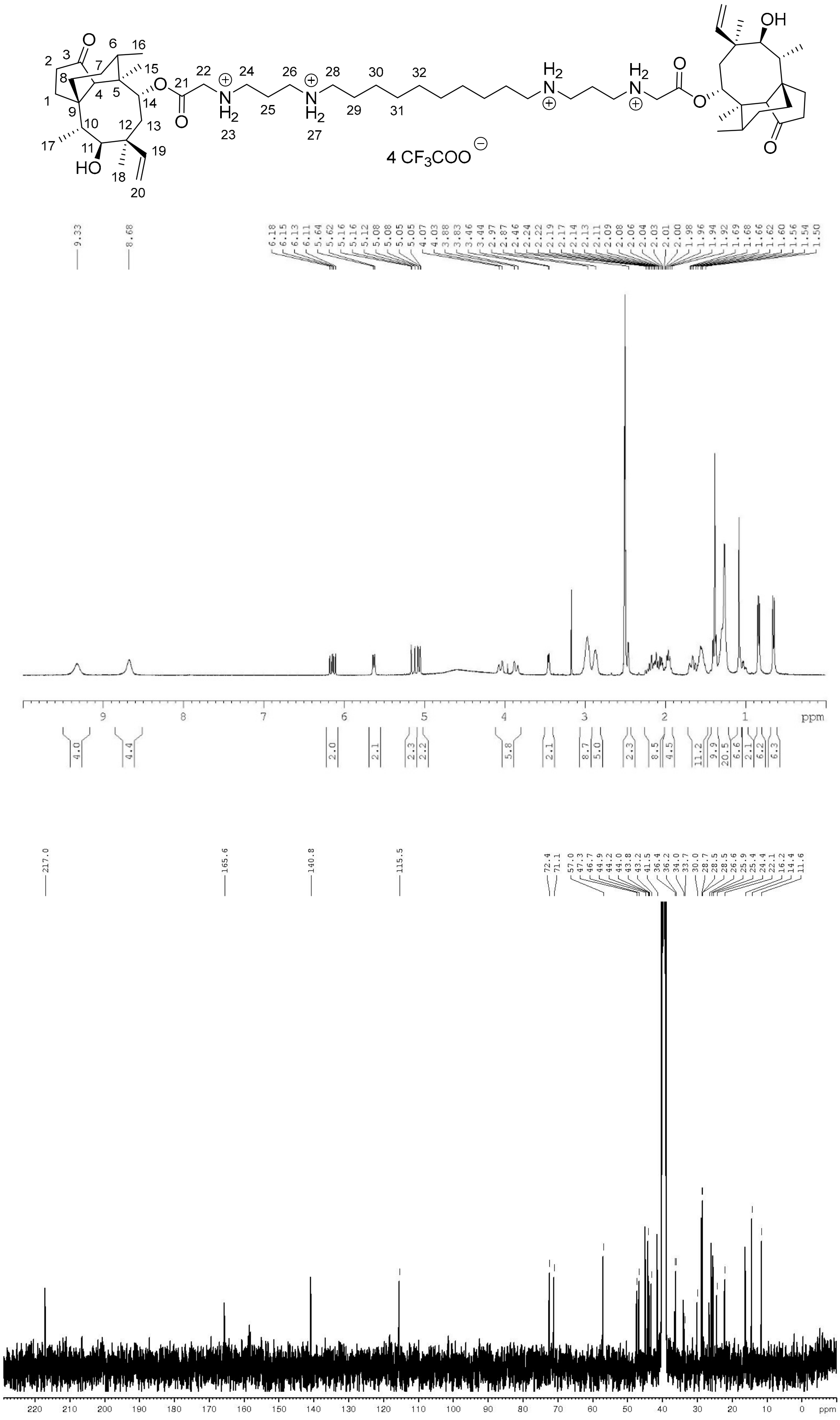

Figure S6 ^{1}H (DMSO-d_6, 400 MHz) and ^{13}C (DMSO-d_6, 100 MHz) NMR spectra for **9e**.

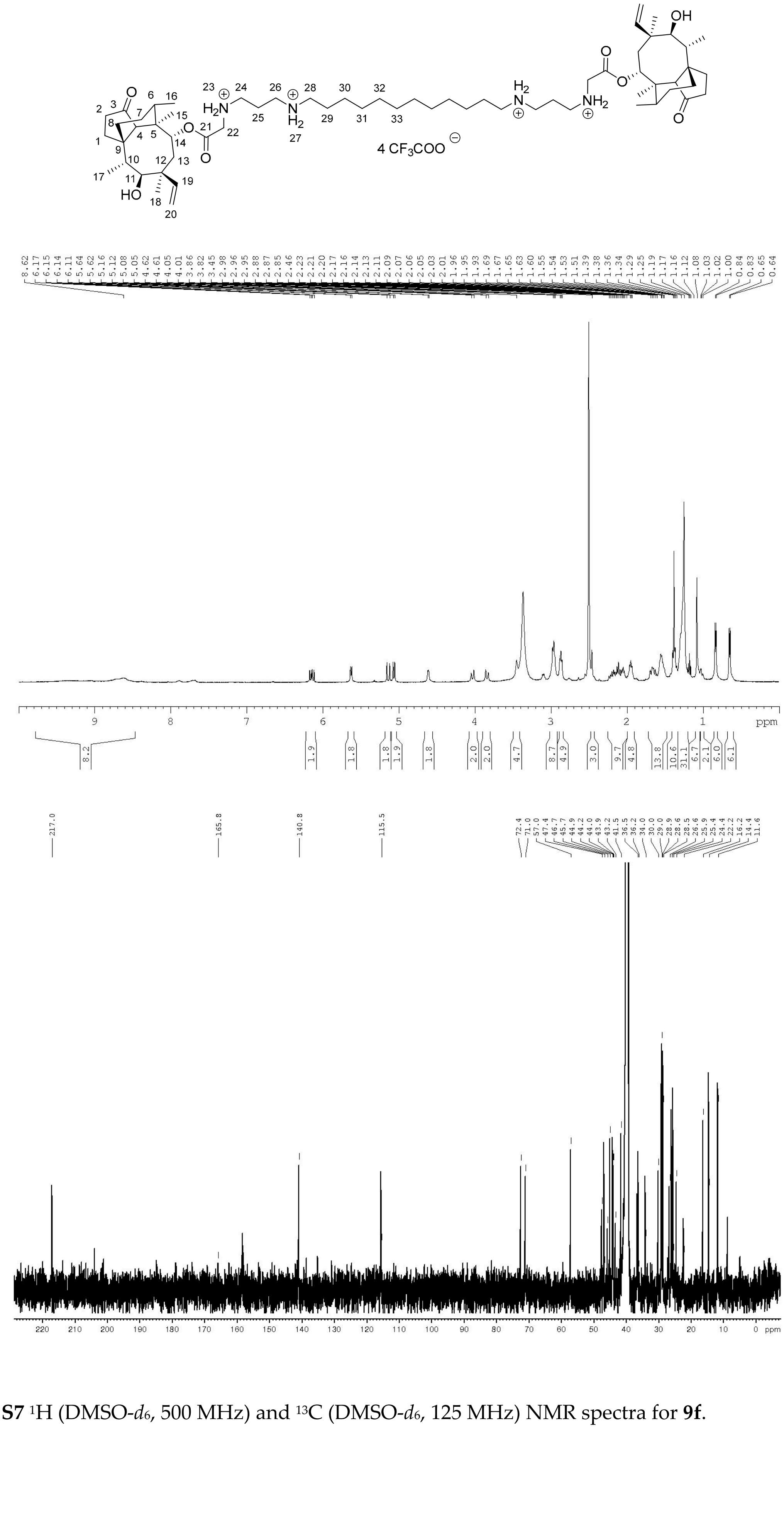

Figure S7 ^{1}H (DMSO-d_6, 500 MHz) and ^{13}C (DMSO-d_6, 125 MHz) NMR spectra for **9f**.

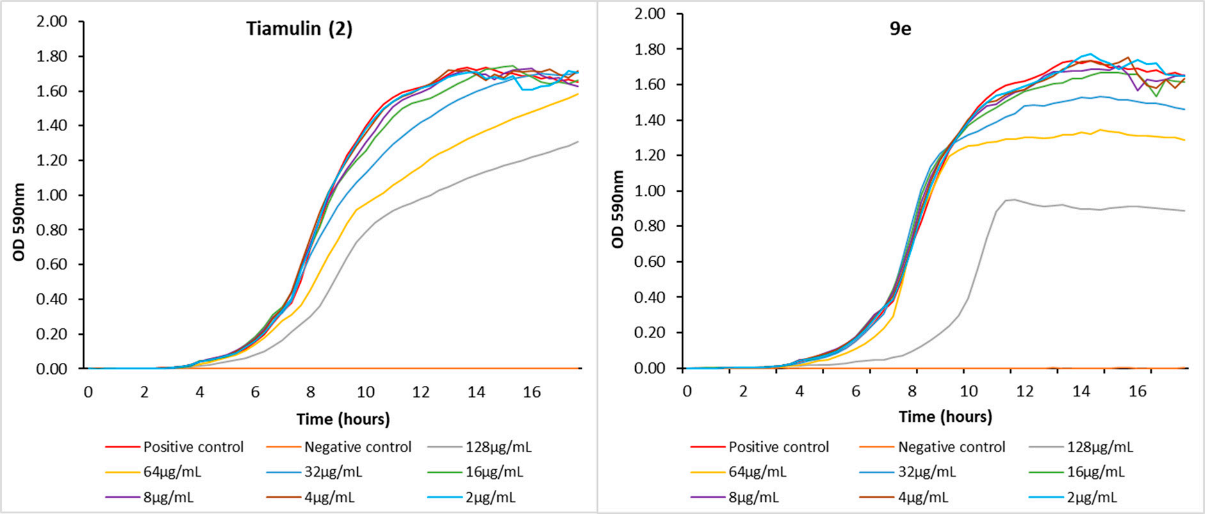

Figure S8 Bacterial growth inhibition exhibited by **2** (left) and **9e** (right) against *P. aeruginosa* PAO1 at different concentrations. Positive control was bacteria only and negative control was media only.
