# Supplementary material for: Preliminary SAR of Novel Pleuromutilin–Polyamine Conjugates
Source: Microorganisms. 2023 Nov 17;11(11):2791. doi: 10.3390/microorganisms11112791 (PMC10673369; doi:10.3390/microorganisms11112791)
Supplement: Supplementary file 1 [file microorganisms-11-02791-s001.zip › microorganisms-2610679-supplemetary-File.pdf]

Supplementary File

Preliminary SAR of Novel Pleuromutilin–Polyamine Conjugates

Kenneth Sue [1], Melissa M. Cadelis [1,2], Kerrin Hainsworth [1], Florent Rouvier [3], Marie-Lise Bourguet-Kondracki [4], Jean Michel Brunel [3] and Brent R. Copp [1,*]

1 School of Chemical Sciences, The University of Auckland, Private Bag 92019, Auckland 1142, New Zealand
2 School of Medical Sciences, The University of Auckland, Private Bag 92019, Auckland 1142, New Zealand
3 Membranes et Cibles Thérapeutiques, INSERM, Aix-Marseille Universite, 27 bd Jean Moulin, 13385 Marseille, France
4 Laboratoire Molécules de Communication et Adaptation des Micro-organismes, UMR 7245 CNRS, Muséum National d'Histoire Naturelle, 57 rue Cuvier (C.P. 54), 75005 Paris, France
* Correspondence: b.copp@auckland.ac.nz

Materials and Methods

2.1. Chemical Synthesis General Methods

Mass spectra were recorded using a MicrOTOF-QII mass spectrometer (Bruker Daltonics, Bremen, Germany) coupled with a KD Scientific syringe pump, with analysis using Bruker Compass DataAnalysis v 4.1 software. Infrared spectra were recorded on a Perkin Elmer Spectrum 100 Fourier Transform infrared spectrometer equipped with a universal ATR accessory. Optical rotations were obtained with a Rudolph Analytical Autopol IV automatic polarimeter using a 0.1 dm cell (concentration units of g/100 mL). All NMR spectra were recorded using a Bruker Avance (Karlsruhe, Germany) AVIII-400 spectrometer operating at 400 MHz for ^{1}H nuclei and 100 MHz for ^{13}C nuclei or AVIII-500 spectrometer operating at 500 MHz for ^{1}H nuclei and 125 MHz for ^{13}C nuclei. Chemical shifts are expressed in parts per million (ppm) relative to the solvent peaks (DMSO-d_6: ^{1}H 2.50, ^{13}C 39.52 ppm; $CDCl_3$: ^{1}H 7.26, ^{13}C 77.16 ppm). Assignments are based on 1- and 2-dimensional NMR experiments and analogue comparisons. Standard Bruker pulse sequences were utilized. Flash column chromatography was carried out using either diol-bonded silica (40–63 μm) (Luknova, Mansfield, MA, USA), Davisil silica gel (40–63 μm) (Merck, Munich, Germany), or C_8 reversed-phase (40–63 μm) (Merck Millipore, Darmstadt, Germany) solid supports. Analytical thin layer chromatography (TLC) was carried out on 0.2 mm thick plates of Merck DC Kieselgel 60 (Munich, Germany) silica F254 or RP-18 F254S plates. All solvents were of analytical grade or better and/or purified according to standard procedures. Chemical reagents used were purchased from standard chemical suppliers (Merck) and used as purchased. Polyamines di-*tert*-butyl butane-1,4-diylbis((3-aminopropyl)carbamate) (**7a**), di-*tert*-butyl hexane-1,6-diylbis((3-aminopropyl)carbamate) (**7b**), di-*tert*-butyl heptane-1,7-diylbis((3-aminopropyl)carbamate) (**7c**), di-*tert*-butyl octane-1,8-diylbis((3-aminopropyl)carbamate) (**7d**), di-*tert*-butyl decane-1,10-diylbis((3-aminopropyl)carbamate) (**7e**), and di-*tert*-butyl dodecane-1,12-diylbis((3-aminopropyl)carbamate) (**7f**) were synthesized according to the literature procedures [22–24].

2.1.1. General Procedure A: Reaction of pleuromutilin 22-OTs (**8**) with nucleophiles

A stirred solution of pleuromutilin 22-*O*-tosylate (**8**) (2 equiv.) and KI (2.2 equiv.) in dry MeCN (1 mL) was heated at 70 °C for 1.5 h under N_2. The corresponding Boc-protected polyamine (1 equiv.) in dry MeCN (1 mL) and DIPEA (6 equiv.) were added to the solution and allowed to stir for a further 2.5 h at 70 °C. The solvent was removed under reduced pressure to yield a crude oil which was dissolved in CH_2Cl_2 (20 mL) and washed with H_2O (5 x 20 mL). The organic layer was dried under reduced

pressure and subjected to silica gel column chromatography (8–10% MeOH/CH_2Cl_2) to afford the desired Boc-protected pleuromutilin intermediate.

2.1.2. General Procedure B: Boc Deprotection

A solution of the *tert*-butyl-carbamate derivative was stirred in CH_2Cl_2 (2 mL) with TFA (0.2 mL) at room temperature under N_2 for 2 h. The solvent was removed under reduced pressure and the crude product purified using C_8 reversed-phase column chromatography (H_2O/MeOH (+0.05% TFA), 100:0⟶3:1) to afford the product as the tetra-TFA salt.

2.3. Antimicrobial Assays

The susceptibility of bacterial strains *S. aureus* (ATCC 25923), *E. coli* (ATCC 25922), and *P. aeruginosa* (ATCC 27853 or PAO1) to antibiotics and compounds was determined in microplates using the standard broth dilution method in accordance with the recommendations of the Comité de l'AntibioGramme de la Société Française de Microbiologie (CA-SFM). Briefly, the minimal inhibitory concentrations (MICs) were determined with an inoculum of 10^5 CFU in 200 μL of Mueller–Hinton broth (MHB) containing two-fold serial dilutions of each drug [26]. The MIC was defined as the lowest concentration of drug that completely inhibited visible growth after incubation for 18 h at 37 °C. To determine all MICs, the measurements were independently repeated in triplicate. Additional antimicrobial evaluation against MRSA (ATCC 43300), *Klebsiella pneumoniae* (ATCC 700603), *A. baumannii* (ATCC 19606), *Candida albicans* (ATCC 90028), and *Cryptococcus neoformans* (ATCC 208821) was undertaken at the Community for Open Antimicrobial Drug Discovery at The University of Queensland (Australia) according to their standard protocols as reported previously [26,27]. For antimicrobial assays, the tested strains were cultured in either Luria broth (LB) (In Vitro Technologies, USB75852), nutrient broth (NB) (Becton Dickson, 234000), or MHB at 37 °C overnight. A sample of culture was then diluted 40-fold in fresh MHB and incubated at 37 °C for 1.5–2 h. The compounds were serially diluted 2-fold across the wells of 96-well plates (Corning 3641, non-binding surface), with compound concentrations ranging from 0.015 to 64 μg/ mL, and plated in duplicate. The resultant mid-log phase cultures were diluted to the final concentration of 1×10^6 CFU/mL; then, 50 μL was added to each well of the compound-containing plates, giving a final compound concentration range of 0.008 to 32 μg/mL and a cell density of 5×10^5 CFU/mL. All plates were then covered and incubated at 37 °C for 18 h. Resazurin was added at 0.001% final concentration to each well and incubated for 2 h before MICs were read by eye.

For the antifungal assay, fungi strains were cultured for 3 days on YPD agar at 30 °C. A yeast suspension of 1×10^6 to 5×10^6 CFU/mL was prepared from five colonies. These stock suspensions were diluted with yeast nitrogen base (YNB) (Becton Dickinson, 233520) broth to a final concentration of 2.5 $\times 10^3$ CFU/mL. The compounds were serially diluted 2-fold across the wells of 96-well plates (Corning 3641, non-binding surface), with compound concentrations ranging from 0.015 to 64 μg/mL and final volumes of 50 μL, plated in duplicate. Then, 50 μL of the fungi suspension that was previously prepared in YNB broth to the final concentration of 2.5×10^3 CFU/mL was added to each well of the compound-containing plates, giving a final compound concentration range of 0.008 to 32 μg/mL. Plates were covered and incubated at 35 °C for 36 h without shaking. *C. albicans* MICs were determined by measuring the absorbance at OD_{530}. For *C. neoformans*, resazurin was added at 0.006% final concentration to each well and incubated for a further 3 h before MICs were determined by measuring the absorbance at $OD_{570-600}$.

Colistin and vancomycin were used as positive bacterial inhibitor standards for Gram-negative and Gram-positive bacteria, respectively. Fluconazole was used as a positive fungal inhibitor standard for *C. albicans* and *C. neoformans*. The antibiotics were provided in 4 concentrations, with 3 above and 3 below its MIC value, and plated into the first 8 wells of column 23 of the 384-well NBS plates. The quality control (QC) of the assays was determined by the antimicrobial controls and the Z'-factor (using positive and negative controls). Each plate was deemed to fulfil the quality criteria (pass QC) if the Z'-factor was above 0.4, and the antimicrobial standards showed full range of activity, with full growth inhibition at their highest concentration, and no growth inhibition at their lowest concentration.

2.4. Determination of the MICs of Antibiotics in the Presence of Synergizing Compounds

Briefly, restoring enhancer concentrations were determined with an inoculum of 5x10^5 CFU in 200 µL of MHB containing two-fold serial dilutions of each derivative in the presence of doxycycline at 4.5 µM (2 µg/mL) and erythromycin 2.7 µM (2 µg/mL) [26]. The lowest concentration of the polyamine adjuvant that completely inhibited visible growth after incubation for 18 h at 37 °C was determined. These measurements were independently repeated in triplicate.

2.5. Nitrocefin assay

Outer membrane permeabilization was measured using nitrocefin as a chromogenic substrate of periplasmic β-lactamase [28]. Ten milliliters of MH broth were inoculated with 0.1 mL of an overnight culture of PAO1 bacteria and grown at 37°C until the OD_{600} reached 0.5. The remaining steps were performed at room temperature. Cells were recovered by centrifugation (4000 rpm for 20 min) and washed once in 20 mM potassium phosphate buffer (pH 7.2) containing $MgCl_2$ (1 mM). After a second centrifugation, the pellet was resuspended and adjusted to OD_{600} of 0.5. Then, 50 µL of the desired compound was added to 100 µL of the cell suspension to obtain a final concentration varying from 3.9 µM to 250 µM. Fifty microliters of nitrocefin were then added to obtain a final concentration of 50 µg/mL. Nitrocefin hydrolysis was monitored spectrophotometrically by measuring the increase in absorbance at 490 nm. Assays were performed in 96-well plates using a M200 Pro Tecan spectrophotometer .

2.6. Cytotoxicity Assays

HEK293 cells were counted manually in a Neubauer hemocytometer and plated at a density of 5,000 cells/well into each well of the 384-well plates containing the 25x (2 µL) concentrated compounds [26,27]. The medium used was Dulbecco's modified Eagle's medium (DMEM), supplemented with 10% fetal bovine serum (FBS). Cells were incubated together with the compounds for 20 h at 37 °C, 5% CO_2. To measure cytotoxicity, 5 µL (equals 100 µM final) of resazurin was added to each well after incubation and incubated for further 3 h at 37 °C with 5% CO_2. After the final incubation, fluorescence intensity was measured as Fex 560/10 nm, em 590/10 nm ($F_{560/590}$), using a Tecan M1000 Pro monochromator plate reader. CC_{50} values (concentration at 50% cytotoxicity) were calculated by normalizing the fluorescence readout, with 74 µg/mL tamoxifen as negative control (0%) and normal cell growth as positive control (100%). The concentration-dependent percentage cytotoxicity was fitted to a dose–response function (using Pipeline Pilot) and CC_{50} values were determined.

2.7. Hemolytic Assays

Human whole blood was washed three times with three volumes of 0.9% NaCl and then resuspended in the same solution to a concentration of 0.5 x 10^8 cells/mL, as determined by the manual cell count in a Neubauer hemocytometer [26,27]. The washed cells were then added to the 384-well compound-containing plates for a final volume of 50 µL. After a 10 min shake on a plate shaker, the plates were then incubated for 1 h at 37 °C. After incubation, the plates were centrifuged at 1,000g for 10 min to pellet cells and debris; 25 µL of the supernatant was then transferred to a polystyrene 384-well assay plate. Hemolysis was determined by measuring the supernatant absorbance at 405 mm (OD_{405}). The absorbance was measured using a Tecan M1000 Pro monochromator plate reader. HC_{10} and HC_{50} (concentration at 10% and 50% hemolysis, respectively) were calculated by curve fitting the inhibition values *vs.* log (concentration) using a sigmoidal dose–response function with variable fitting values for the top, bottom, and slope.

2.8. Real-time growth curves

Solutions of the compound at concentrations of 2, 4, and 16 µg/mL were tested each in triplicate against *S. aureus* ATCC 25923 [26]. Typically, in a 96-well plate were placed 10 µL of 40, 80, and 320 µg/mL stock solutions of compound, as well as 190 µL of a 5 x 10^5 CFU/mL of the selected bacterial suspension in brain heart infusion (BHI) broth. Positive controls containing only 200 µL of a 5 x 10^5 CFU/mL of bacterial suspension in BHI and negative controls containing only 200 µL of BHI broth were added. The plate was incubated at 37°C in a TECAN Spark Reader (Roche Diagnostic) and real-time bacterial growth was followed by OD_{590} nm measurement every 10 min during 19 h.

2.9 ATP efflux measurement

A solution of the compound was prepared in twice-distilled water at a fixed concentration of 100 μg/mL. The different Gram-positive suspensions were prepared in MH II broth and were incubated at 37 °C. Then, 90 μL of each bacterial suspension was added to 10 μL of the compound solution and shaken for 20 s in the incubator at 37 °C. Subsequently, 50 μL of Luceferin–Luceferase reagent (Yelen, France) was added to the mixture, and the luminescent signal was quantified with an Infinite M200 microplate reader (Tecan, Männedorf, Switzerland) for five seconds [28]. ATP concentration was quantified by internal sample addition. Squalamine (100 μg/mL) was used as a positive control to quantify the maximum level of ATP efflux and water as the negative control. This assay was performed in three independent experiments.
